# Posture Affects How Robots and Infants Map Words to Objects

**DOI:** 10.1371/journal.pone.0116012

**Published:** 2015-03-18

**Authors:** Anthony F. Morse, Viridian L. Benitez, Tony Belpaeme, Angelo Cangelosi, Linda B. Smith

**Affiliations:** 1 Cognition Institute, Center for Robotics and Neural Systems, University of Plymouth, Drake Circus, Plymouth, PL4 8AA, United Kingdom; 2 Department of Psychology, University of Wisconsin-Madison, 1202 W. Johnson St., Madison, WI 53706, United States of America; 3 Psychological and Brain Sciences, Indiana University, 1101 East Tenth Street, Bloomington, IN 47405-7007, United States of America; Lancaster University, UNITED KINGDOM

## Abstract

For infants, the first problem in learning a word is to map the word to its referent; a second problem is to remember that mapping when the word and/or referent are again encountered. Recent infant studies suggest that spatial location plays a key role in how infants solve both problems. Here we provide a new theoretical model and new empirical evidence on how the body – and its momentary posture – may be central to these processes. The present study uses a name-object mapping task in which names are either encountered in the absence of their target (experiments 1–3, 6 & 7), or when their target is present but in a location previously associated with a foil (experiments 4, 5, 8 & 9). A humanoid robot model (experiments 1–5) is used to instantiate and test the hypothesis that body-centric spatial location, and thus the bodies’ momentary posture, is used to centrally bind the multimodal features of heard names and visual objects. The robot model is shown to replicate existing infant data and then to generate novel predictions, which are tested in new infant studies (experiments 6–9). Despite spatial location being task-irrelevant in this second set of experiments, infants use body-centric spatial contingency over temporal contingency to map the name to object. Both infants and the robot remember the name-object mapping even in new spatial locations. However, the robot model shows how this memory can emerge –not from separating bodily information from the word-object mapping as proposed in previous models of the role of space in word-object mapping – but through the body’s momentary disposition in space.

## Introduction

Robotics makes clear the evolutionary feat that is biological intelligence [1–4]. Smooth and effective action in a constantly changing physical world requires the continuous coupling of sensors and effectors to those changing physical realities [2,5–8]. However, an intelligent system that does more than react also needs stable cognitive products such as categories and decisions that are at least partially decoupled from the here-and-now on which sensing and acting are so dependent [9]. Building artificial devices that can perform both sensorimotor and cognitive tasks—for example, walk up hills *and* learn language—has not yet been achieved [10,11]. One relevant debate in the study of both artificial and biological intelligence is how cognitive and sensory-motor components should relate to each other. One possibility, sometimes known as the cognitivist solution [12], is that cognition receives information from the sensors and passes information to effectors but is fundamentally distinct and separate from sensorimotor processes in the symbolic character of its cognitive computations [13–15]. The alternative possibility, sometimes known as the embodiment solution [1,7,8] is that there are no distinct computational principles for cognition versus perception and action and that, instead, cognitive processes emerge out of and are dynamically coupled to sensorimotor systems [1,6,16–22]. In this view, cognitive products, although partially decoupled from the here-and-now, are realized in and through the same sensory-motor systems involved in action and perception [21]. Although this second approach seems to promise a smooth integration of cognitive decisions with perception and action, there are many open questions about just how such an “embodied” solution would actually work to solve any specific cognitive task [23–25].

Here we provide an example of an embodied solution to the task of mapping heard names to seen things. Our novel solution integrates cognitive and sensorimotor processes by using bodily posture as the frame of reference for both planning actions and mapping names to objects. Within this cognitive architecture, the internal representations of objects and names are dynamically bound to the body’s momentary disposition in space; nonetheless, performance has a “cognitive” signature in that the robot can recognize a learned name for an object in new postural positions. We also show that this solution may be relevant to biological forms of intelligence by testing novel predictions from the robot model with 16-month-old human toddlers.

The problem we consider is how a naïve learner, an infant, might map a name to an object and then later show memory for that mapping. This mapping problem has generated considerable theoretical interest [26,27] as everyday learning contexts contain multiple objects and unknown names. There are several cognitive solutions in which objects and names are represented as mental entities independent of their experienced spatial relation to the body [26–28]. However, in building a robot model, the body cannot be ignored; for a physically-realized learner to learn anything at all from real time experiences with words and objects, the body must, at the right moment, turn its sensors to the referred to object. Because sensors are in the body, the body—and its spatial disposition—is essential to the *initial* mapping of the name to a thing. But is the body relevant to more than this initial mapping? A growing body of research with infant learners [29–31] suggests that the spatial consistency of the direction of attention to the name, to the object, and across repeated encounters with the name and object increases the likelihood of mapping the name to the object and remembering that mapping. One account of these findings, the Dynamic Neural Field model (DNF) [31] binds features by virtue of their shared spatial position (without specification of the spatial frame as body-centric or allocentric) and then projects the result of this binding to a second field that represents words and objects but has no spatial component. Thus, the DNF learns words and objects through spatial correspondence but the specific learned mapping between words and objects remains independent from spatial information. The model as a whole system is still able to demonstrate spatial biases because object features, primed by words, project back into the object-space map which contains memories of the locations where those object features have been previously encountered. We propose an alternative account that was motivated by the task of building a physical device that can map words to objects and then show through physical behavior that it has remembered that mapping. The robotic model maps words—as does the DNF– through space, but in the robotic model those spatial representations are body-centric and always tied to the momentary posture of the learner. The robotic model generalizes these learned mappings to new spatial locations despite the through-body, and thus indirect, mapping between words and objects. In this model; words, objects, and their remembered associations are always *through* posture and hence are an explicit example of learned sensorimotor knowledge. Finally, this robotic posture model makes novel predictions not made by the DNF that are tested and confirmed in young children.

## Robot Results

The goal of building a physical learner makes clear how the here-and-now of space and the learner’s position in space are essential both to learning from the environment and to demonstrating any of that learned knowledge through behavior. Motor plans for any goal-directed body movement starts with the body’s current position. If the learner has an articulated body capable of many different kinds of fluid actions, with many degrees of freedom, then there are many bodily starting positions and many different ways of moving from any of those starting positions toward the named entity. This body-space problem is fundamental to all motor behaviors [32]: In brief, the local realities of a physical body in space complicate the mapping of names to things and highlights the theoretical problem of how mental entities such as object and name representations that seem to have no spatial or bodily components interact with processes of perception and action.

**Fig 1:**
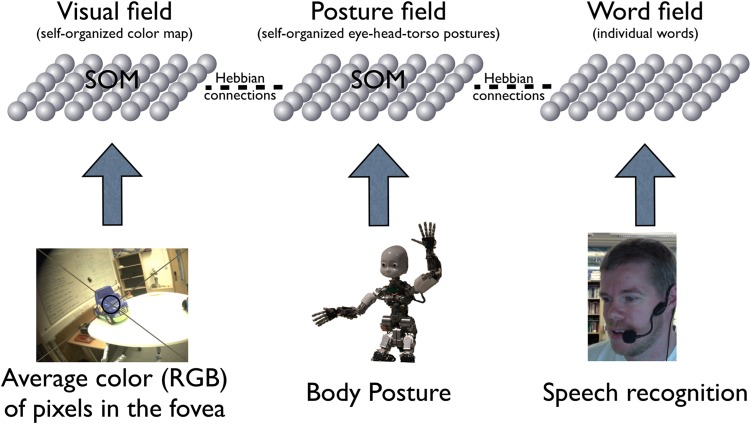
The neural model controlling the iCub robot in ongoing learning. External input to each field is constantly driven by visual input, momentary body posture, and online speech recognition. Internal input to each field is a spreading activation via associative connections subject to ongoing learning and via the body posture. Note: the neural model forms the highest layer of a subsumption architecture controlling the robot, further details are in the Supplementary Information to this paper. (The individual shown in this figure has given written informed consent (as outlined in PLOS consent form) to publish this image).

To address this fundamental theoretical problem, our cognitive architecture used information about the body’s posture (joint angles of eyes, head, and torso) for planning actions and for linking the internal activations generated by heard names and seen objects to each other both at initial learning and at the recall of that information. The neural model was instantiated in a humanoid iCub robotic platform [33] as a network of associations formed directly (and continuously) between body posture information and separate streams of sensory information. Body posture information therefore formed an intermediate ‘hub’ connecting the multimodal information about names and objects (see Fig. 1).

The implemented model used a Word Field, a Visual Object Field, and a Posture Field each consisting of a population of interconnected “neuron-like” elements and each continuously driven by raw sensory input and forming memories through self-organized learning [34], or in the case of the Word Field a one-to-one binding with the products of speech recognition. Activity spread via the Posture Field among all three fields so that activation in any field influenced (by priming and/or inhibiting) the pattern of activation in other fields. Change or movement saliency in the visual field caused the robot to orient to the location of that change; primed visual features (via activation in another field) similarly provoked bodily orientation to the primed objects. The linked fields continuously adapted, altering the strength of connections within and among the fields via ongoing unsupervised learning (equations in S1 File) [35]. Critically, there was no direct connection between the Word and Visual Object Fields; these were linked only indirectly through the Posture Field, which thus served as the spatial index for both experienced words and objects. Because the fields form memories and because they are connected to each other, sensory input and activation in one field influenced the activation in other fields in much the same way as connectionist spreading activation models [36,37].

**Fig 2:**
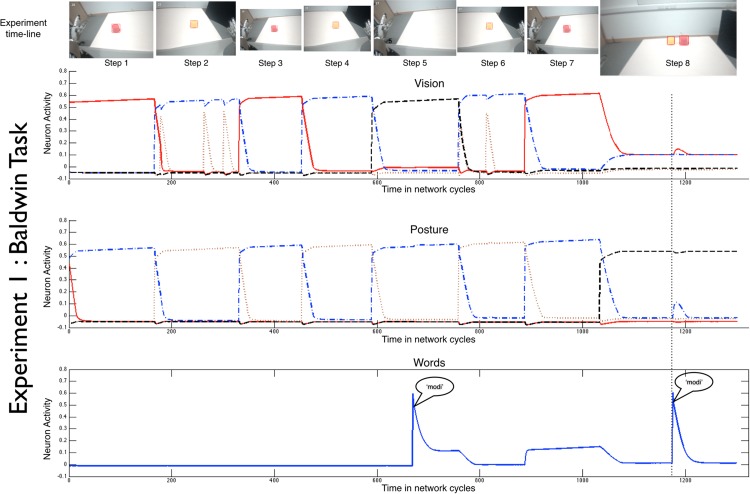
The timeline of an individual in Experiment 1 (no-switch condition), showing the neural activity in the Vision, Posture, and Word Fields as well as the visual input to iCub at each step.

In Experiment 1, the robot’s ability to map names to objects was tested in the Baldwin task [38] in which toddlers have been shown to map a heard name to a seen object even when those two sensory events were separated in time but aligned through the spatial orientation of the body [29,31]. There were 8 steps in our version of the original Baldwin task (see Fig. 2, for a discussion of the neural activity see S1 Fig. and S2 Fig.). In Steps 1 to 4, two objects were presented; one designated the target object (to be associated with the name) and the other the foil.

The robot was first shown one object (the target) on a table 25cm to one side of midline (e.g., left) so that the robot had to turn and look down to see the object (Step 1). The robot was then shown the foil object on the table 25cm to the other side of midline (e.g., right) so that the robot positioned its body differently to view the foil (Step 2). These two presentations were repeated (Steps 3 and 4). This series of presentations created activations (and thus associations) between the visual properties of the objects and the body’s momentary posture (Fig. 2B-C). In Step 5 *with no object in view*, the experimenter directed the robots attention (and elicited a posture) associated with the location of the target and said the new word “modi”. This input drove activation in the Word and Posture fields and constitutes a potential mapping of the heard name to the object, albeit indirectly through the associated activations in the Posture Field. In Steps 6 and 7, the two objects were presented at their associated locations without naming. Step 8 was the test of Word-Object mapping; the two objects were presented in novel and shared locations to which the robot oriented. External Visual input was then briefly stopped, the word ‘modi’ was spoken, and both the behavioral (orientation to and reaching to the target object) and internal response of the robot was measured. Although the stopping of visual input at this point is unnatural (and not what would happen with a living learning), we did it to control for a potential bias resulting from the varying exposure time to each object with the goal of isolating posture as the controlling variable. An internal activation of the perceptual properties associated with the target requires that the neural model map the name to the object through the shared association with a posture (a sensory motor solution). However, because we test that mapping in a new location, that mapping must transcend momentary posture (a seemingly cognitive solution), which the robot results show can be obtained even though the posture field is the only internal link between word and object during both learning and test.

Across 20 independent runs of the experiment (with all connections between fields reset to zero, and both SOM’s weights reset to random values between simulations), the robot behaviorally indicated knowledge of the link between the object and name on 71.25% of tests (t(19) = 2.28, p < 0.05, d = 0.51) (see S3 Fig. for the measures of the strength of internal associations among postures, words, and associations). Experiment 1 (Baldwin task) shows how sensorimotor representations that are tied to the postural orientation of the body (and thus are smoothly driven by and drive action) can yield knowledge with one signature character of cognition: seeming non-dependence on the body’s momentary posture. But in this cognitive architecture, that knowledge emerges through and is tied to representations of posture.

Experiments 2 (switch task) and 3 (posture change) showed the critical role of posture in two ways. In Experiment 2 (S5 Fig., S6 Fig., and S7 Fig.), the object locations were reversed in Steps 1 and 2 only (see [31] for a parallel experiment with children) so that there was not a distinct posture associated with the target and foil. Again, there were 20 independent runs using this procedure. As predicted, and as observed in a previous study with children (31), this manipulation of changing spatial positions resulted in poorer performance by the robot than was obtained under the spatially consistent presentation of objects in the baseline procedure of Experiment 1 (Baldwin task) (p < 0.05). The robot failed at test to consistently orient and reach to the target referent, choosing the target on only 46% of the tests, which did not differ significantly from chance (p = 0.64). The robot failed because the target and foil were not associated with distinct postures, providing no way to map internal representations of the target, rather than the foil, to the name. Experiment 3 (posture change) (see S8 Fig., S9 Fig. and S10 Fig.) disrupted learning by removing the shared associated posture between the target and the heard name. The timeline of events was the same as in Experiment 1 (Baldwin task) with one exception: the robot’s posture during the naming event (Step 5) differed from all other events. For example, if the robot was standing for Steps 1–4 and for steps 6 to 8, the robot was instructed to sit down for the entirety of Step 5 (see S8 Fig.). This shift in posture should disrupt the linking of the target to the heard name. Consistent with this prediction, across the 20 independent runs of Experiment 3 (posture change), the robot performed more poorly than given the consistent posture procedure of Experiment 1 (Baldwin task), (p < 0.05), failing to consistently orient and reach for the named target at test (p = 0.85), (see S10 Fig. for measures of internal activation). The robot failed to make the mapping because in this cognitive architecture, linking activations in two sensory fields required overlapping postural representations. This fragility of mapping words to objects when the two sensory events are experienced in different postures might be considered a limitation in an abstract cognitive system. However, in a physical world, the components of the same meaningful event may be typically experienced from the same postural perspective and thus bodily changes help segment events that should not be bound together.

**Fig 3:**
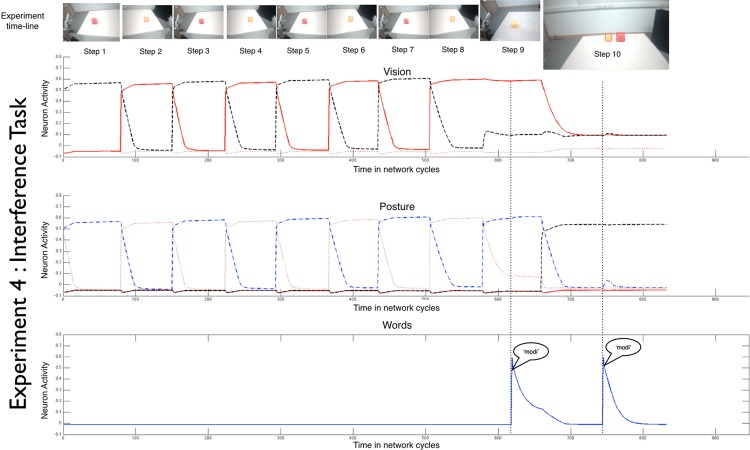
The timeline of an individual in experiment 4 (interference task), showing the neural activity in the Vision, Posture, and Word Fields as well as the visual input to iCub at each step.

Experiments 4 (interference task) and 5 (interference with posture change) made this point by showing how the robot’s architecture may usefully reduce interference between two events that are about different objects. The timeline of events in Experiment 4 is shown in Fig. 3 and is based on an Interference Task first used with children [31]. Here, the target is in view during the naming event (Step 9) but its location (and the elicited posture) was associated with the foil during Steps 1–8. This sets up two strong but competing spatial indices for mapping the name to the object—the past history of association of direction of attention with the foil, and the temporal and spatial consistency of object and word during the naming event (see S11 Fig., S12 Fig., and S13 Fig. for the resulting competing activations in the Visual Field). The procedure of Experiment 4 (interference task) should lead to poor performance in the robot as it does in children (31). We explicitly tested the role of posture in reducing interference in Experiment 5 (interference task with posture change). The timeline of events was the same as in Experiment 4 (interference task) with one exception: the robot’s posture during the naming event (Step 9) differed from all other events (see S14 Fig.). This one shift in posture should weaken the interfering activation from the previous association of the foil with that location.

In 20 independent runs of the Experiments 4 (interference task) and 5 (interference task with posture change), the robot, as predicted, performed better given a shift in posture between the interfering events, (p < 0.05). In Experiment 4 (interference task) when the foil was associated with the posture of the naming event, the robot chose the target on 42% of the tests (see S13 Fig. for measures of internal connections); in Experiment 5 (interference task with posture change) when a posture shift was instituted during the naming event, the robot chose the test, 61% of the tests, (see S15 Fig. for measures of internal activation). In neither experiment did performance differ from chance (p = 0.85 and p = 0.13, for the two experiments respectively) however, performance between these two experiments was significantly different (p < 0.05). The high variability in robot performance across different runs in these two experiments is understandable—and potentially meaningful—on two grounds. First, the visual input as well as body position in a physical world is inherently variable. Second, the interference condition sets up two strong and competing links through which the robot could bind a name to a thing. In both Experiments, small variations in physical reality can lead to different resolutions of this competition, a fact potentially informative to the high level of variability often seen in children’s performances.

S17 Fig. summarizes the robot experiment results achieved across each condition showing both the results achieved for a low learning rate of 0.012 and also for a higher learning rate of 0.1, demonstrating that the qualitative differences between each experimental condition remain robust across these parameter changes.

## Infant Results

**Fig 4:**
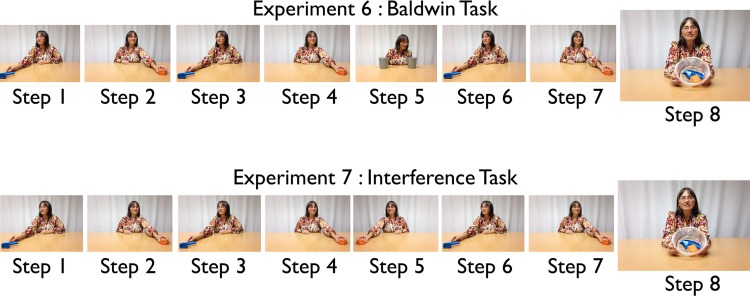
Timeline of experiment 6 (above) and experiment 8 (below). Steps 1–4 expose the infant to the target and foil objects in consistent left and right locations. In step 5 the infant is told ‘this is a modi’ while the objects are out of sight (hidden in buckets) in experiment 6, or while the foil object is in the target object location and being attended in experiment 8. Steps 6 & 7 repeat the original exposure of the target and foil, and in step 8 the infant is shown both objects in a new location and asked ‘where is the modi’. Experiments 7 and 9 follow the same timeline with the addition that step 5 occurs in a different posture from all other steps. (The individual shown in this figure has given written informed consent (as outlined in PLOS consent form) to publish this image).

Studies of word-object mapping by 1–1 1/2 year old infants, including variants of the Original Baldwin task and the Interference task [29,31,38] show that the spatial consistency of experienced names and words are critical for learning. However, there are several possible explanations of why spatial consistency matters for early learning (see, [29]), and the role of posture, the body’s momentary disposition in space as it orients to events in the world, has never been directly tested. Accordingly, Experiments 6 to 9 were replications of the Robot experiments with toddlers: Experiment 6—Original Baldwin task with no posture shift (Robot Experiment 1), Experiment 7—Original Baldwin task with posture shift at Step 5 (Robot Experiment 3), Experiment 8—Interference task with no posture shift (Robot Experiment 4), and Experiment 9—Interference task with posture shift at Step 5 (Robot Experiment 5). The timeline of events for the infant experiments are shown in Fig. 4.

**Fig 5:**
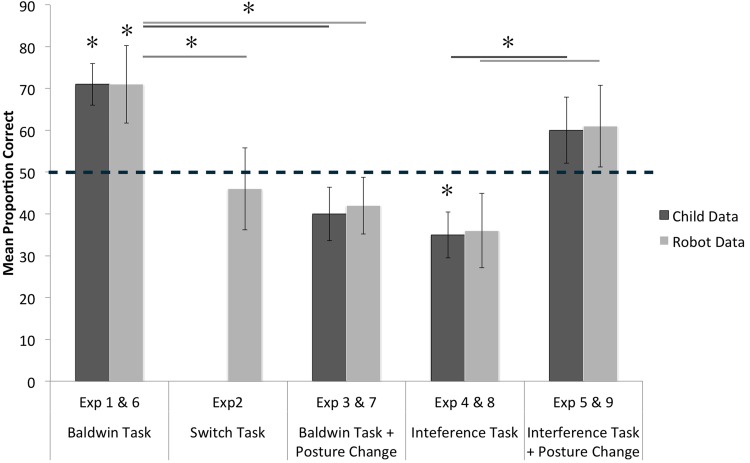
Comparison between the Child and Robot data showing the means of the proportion of correct choices (and standard error of the means) for all experiments, and using the low-learning rate robot data. Dotted line denotes chance, p < 0.05. Specific values for the child data and the robot data is as follows: For the Original Baldwin task, when objects and names were separately linked to the same posture, the robot correctly mapped the name to the target (Exp1), M = 0.71 (SD = 0.41), at above chance levels, t(19) = 2.2, p < 0.05, d = 0.51. Infants also correctly mapped the name to the target (Exp6), M = 0.71 (SD = 0.20), at above chance levels, t(15) = 4.16, p < 0.001, d = 1.04. In Experiment 2, where the locations of objects was switched, the robot failed to map the name to the target, M = 0.46, p = 0.64, but did so reliably less often than in the standard Baldwin condition t(38) = 0.03, p < 0.05, d = 0.58. In the Baldwin task with posture change, when Step 5, the naming event, was experienced in a new posture, the robot (Exp3) and the infants (Exp7) failed to map the name to the object, both preforming at chance Robot; M = 0.42, p = 0.85, Child; M = 0.41, p = 0.16, and did so reliably less often than in the standard Baldwin Task where there was no posture shift; Robot; t(38) = 2.49, p < 0.05, d = 0.78, Infant; t(30) = 3.73, p < 0.001, d = 1.32. In the Interference task, the toddlers showed the same interference effect as the robot, and as the toddlers in Samuelson et al., 2011; when the target object was explicitly named at a location and posture associated with the distractor object, both the robot (Exp4) and children (Exp8) selected the target referent at *below* chance levels however only the child data was significantly below chance, M = 0.36 (SD = 0.4), t(19) = -1.5, p = 0.07, d = 0.34, Robot data p = 0.07. For the Interference task with a posture change, when the Phase 1 experiences were distinguished from the Phase 2 naming events by a poster shift, although performance was not above chance, both children (Exp9) and the robot (Exp5) the interference effect present in Experiment 4 & 8 was reduced p = 0.09 & p = 0.13 respectively. However for both child data and robot data the named target in the posture shift condition was reliably selected more often than when there was no posture shift Child; t(30) = -2.59, p < 0.05, d = 0.91, Robot; t(38) = -1.87, p < 0.05, d = 0.24.

The results (Fig. 5) replicated the qualitative findings with robots: Postural consistency enabled infants to link a name and object experienced at distinct times but created interference when different meaningful events (different objects) are associated with overlapping postural stance. However for Experiment 8 (interference task), in which the target is named in the foil location associated with a different posture, infant results show a significantly below chance level of selection of the target, while in the robot experiments this is not significantly below chance. This suggests that infant memory for the object previously associated with a postural direction of attention is stronger than was assumed in the robot model. The key point, however, is this: Although posture is not typically considered a relevant factor in human cognition (but see [39–42]), humans, like the robot, are faced with the problem of how to integrate cognition with bodily actions that always depend on the momentary posture and thus one expedient solution—for toddlers as well as the robot model—may be the dynamic binding of cognition to body posture. The infants, like the robot, were tested for their word-target mappings in new locations and postural stances. Thus, the infant results, like the robot model, indicate that sensorimotor representations tied to postural orientation (and thus smoothly linked to bodily action) can yield one signature character of cognition: the *seeming* independence of knowledge from the body.

Directly comparing the robot and child data (see Fig. 5) we can see a very close match between the overall performance for the children and the low-learning-rate robot in each condition (within 2%) and yet there are differences: on the interference task with no posture shift, the child data (experiment 8) was significantly below chance while the robot data (experiment 4) was not. In every experimental condition we can see that the standard error is slightly greater for the robot vs child data, showing that the robot data was slightly more variable than the child data. We attribute both differences to inherent noise in the camera image driving the visual system leading to variation in the object boundaries and hence variation in the color information driving the color SOM. The key result is the similar qualitative pattern for the robot and the children, a pattern that implicates a role for momentary posture in young children’s mapping of a name to an object.

## Conclusion

The link between posture and mapping in the robot model and in the infants challenges theories that disassociate learning and memories for words and objects from the body and its disposition in space. The robot model is also an advance over the DNF model on the role of spatial consistency [31] because that model makes no prediction about posture as it does not specify the relevant spatial frame. The robot model—in the service of a physical body that must acquire and demonstrate information in space—proposes label-to-posture and object-to-posture associations, and thereby not only specifies the relevant spatial frame, body posture, but makes it central to learning. The infant results support this position; young learners, who also have bodies in space, exhibit learning patterns like the robot that are tied to the body’s position in space. This link between mapping labels to objects via posture in infants has potentially far reaching implications. Many atypical patterns of motor development are co-morbid with cognitive developmental disorders [43–45]. The link between abnormal movement patterns and poor attentional control in children, poor language learning, and at risk cognitive development (see, [45,46]) are well-known but not well understood. The present model provides hypotheses toward a mechanistic understanding of the developmental dependencies between sensory-motor processes and early cognitive development in that the coordination of the body in space plays a role in binding multimodal events and in retrieving those memories.

One open question given the present results concerns development. Is the tie to posture for infant learning a fact about infants or a general fact about how the brain and how its functional networks extend into the body [47]. One possibility is that the findings are about early infant learning and perhaps related to the immature hippocampal system [48]. Another possibility is that the link between memories and the body found here is generally true, even in adults, albeit perhaps not so easily shown. First, the spatial frames of reference for eye, head, trunk, and hand [49–53] interact and bias each other suggesting a role for overall posture in the bodily frame of reference. Second, localized spatial attention plays a fundamental role in binding sensory information in adults [e.g. 54,55] and the brain networks that underlie localized attention overlap with the networks underlying the spatial frames for bodily action [56–61]. In sum, future work is needed to determine whether a role for posture in mapping words to objects is specific to and perhaps diagnostic of an immature learning system or whether it reflects the more general principle of how cortical networks extend to the sensory-motor surface [47].

The robotic model used the bodily frame of reference that is necessary for planning actions to also integrate information across space, time, and modalities, an expedient engineering solution that may also have been discovered through biological evolution. This solution made novel and confirmed predictions about the role of posture in infant learning. There are other ways to explain why posture shifts have the experimental effects they do [see 62]. The posture solution, however, derives from evidence and theoretical principles concerning the centrality of the body’s posture to all sensory-motor functions. Momentary posture determines the nature and structure of both input and planned actions, and in so doing provides a continuous context through which sensory-inputs gain their meaning.

## Method

### Robot method

100 randomly initialized robot models (further details in S1 File) participated with equal numbers assigned to each of the 5 robot experiments. The two objects were a red sensory ball, and a yellow plastic cup. Each object was between 7–9cm in length and width and height. Each served as the target referent for half the robot models in each experiment. In Experiments 1 (Baldwin task) and 2 (switch task), the robot could see the white surface of the table during the naming event. In Experiments 1 (Baldwin task) and 4 (interference task), half the robot models were in a seated position through out the experiment and half in an upright standing position. In the Posture Change procedures of Experiments 3 (posture change) and 5 (interference task with posture change), half the robot models sat during Phase 1 (Object presentation), then stood at Phase 2 (Naming); the other robot models, stood at Object presentation then sat at Naming. In each trial of all experiments each individual network was tested 4 times. A video camera recorded the interaction for the later coding. A video of the interaction with the robot for experiments 4 and 5 is included in the supplementary information for this paper (S1 Video).

### Infant method

64 toddlers, half male, within +/- 3 weeks of 16 months participated with equal numbers assigned to each of the 4 Experiments (6–9) (6 additional children were recruited but did not contribute data due to shyness, poor performance on control objects at test as described below, or experimental error). Parents of all child participants provided written informed consent prior to the experiment. All experimental protocols and the consent materials were approved by the Indiana University Institutional Review Board. Children in all experiments were from monolingual middle-class homes in a Midwestern town. Participant names were obtained from public birth announcements.

The two objects were a transparent cube with moving colored beads inside and a bright blue plastic toy garlic press that opened and closed. These were selected through extensive pilot testing such that infants demonstrated no significant preference for one toy over the other. For half the infants in each experiment the garlic press was the target and for the other half the cube was. Each object was between 5–7cm in length, 4–7 cm in height, and 4 cm in width. Each served as the target referent for half the children in each experiment. In Experiments 6 (Baldwin task) and 7 (posture change), two identical grey plastic buckets, 15 cm high with a diameter of 12 cm, were also used to hide the two objects during the naming event. For testing of children’s knowledge of the name-referent mapping, a small transparent container (10 X 10 cm, 5 cm high) was also used. In addition, small toys, typical instances of a banana, spoon, duck, and cat, were used as warm up and control test items.

In the no-posture shift procedures of Experiments 6 (Baldwin task) and 8 (interference task), half the children sat on their parent’s lap through out the experiment and half stood on the parent’s lap; in both cases parents’ supported the trunk with the their hands. In the Posture Change procedures of Experiments 7 (posture change) and 9 (interference task with posture change), half the children sat during Phase 1 (Object presentation), then stood at Phase 2 (Naming), then sat for Phase 3 (Object re-presentation); the other children, stood at Object presentation, sat at Naming, then stood for the Object re-presentation phase. All children sat during the Test. In all conditions, the mother was instructed to hold her child—with both hands—at the waist, and other than to help her child stand or sit as instructed, not to participate in the experiment and explicitly not to touch, mention or motion any of the toys, nor to help the child in anyway. A video camera recorded the interaction for the later coding and to affirm parental compliance.

Experiments 6 (Baldwin task) and 8 (interference task) followed the original procedure in the Baldwin task used with toddlers [35] and the results in the Constant Posture procedure of Experiment 6 constitute a replication of those previous findings. The experiment began with warm-up trials (with the child in the posture assigned for the initial trials). The warm-up trials were designed to familiarize the child with the testing procedure used at the end of the session, and to familiarize the child with interacting with the experimenter. The experimenter presented the child with two familiar objects (e.g., banana and kitty) and told the child the names (“See this banana? Look, here is a kitty”). The experimenter then put the objects in the test container and told the child to get one object, e.g., “Get the banana.” Correct choices were cheered and incorrect choices were corrected. This was repeated until the child correctly indicated the requested object on 3 consecutive trials. Phase 1 of the Experiment with the novel objects began immediately. The target object was presented first, either 25 cm to the right or 25 cm to the left of midline (counterbalanced across children). The experimenter held the object up to the right or left of midline, saying “Look at this. See this” for 5s while performing an action with it (shaking the cube or opening and closing the toy garlic press), and then placing it on the table at the side it was presented on, following an imaginary line approximately 25 cm off midline so that the child looked and reached to the object on that side. After the child examined the object for approximately 5s, the experimenter took it back, again moving it along the same imaginary line on which it had been presented. The experimenter then presented the distractor object 25 cm off midline on the opposite side and repeated the procedure 3 more times, making a total of 4 presentations for each object. The target and distractor objects were always presented on opposite sides, but at a consistent side for each object (e.g., for one child, the cube was presented always on the left, and the garlic press always on the right). For Phase 2, Naming, the experimenter placed the target and distracter objects in separate buckets, out of view of the child. The buckets, with the toys inside, were placed on the table, one 25 cm to the right of midline, the other 25 cm to the left such that the bucket containing the target was on the same side as the presentation of the target during the familiarization phase. The bucket containing the distracter object was on the opposite side, again remaining consistent with where the side the distractor object was presented on during the Naming phase. Next the experimenter tapped the bucket with the target object, and said “modi” three times, while looking straight into the child’s eyes. Following the naming event, for the Object re-presentation phase, the Experimenter gave the child first the distractor, with no naming, following the procedure in Phase 1, and then the target. This imposes a delay of about 10–15 sec between the naming event and the testing. The test trials were structured identically to the warm-up trials but without feedback. Both objects were placed in the same transparent container at midline, with no consistent spatial arrangement, and the child was asked to “Get the modi.” During this period, the experimenter maintained her gaze directly at the child’s eyes. Four test trials with the target and distractor were alternated with 4 control trials in which children were asked to select between pairs of familiar toys previously seen in the warm-up. These were included to maintain interest, to break up the requests for the target, and to insure children understood the task. Children who failed to select the correct objects on 3 of 4 control trials were not included in the final sample. The task took approximately 15 minutes. During the procedure, one video camera was focused on the child and parent. Performances were scored offline from the video. The procedure in Experiments 8 (interference task) and 9 (interference task with posture change), was identical except for Phase 2, the Naming event. Here the target object was placed in full view on the table 25 cm to the right or left of midline and on the *opposite* side from its location during Phase 1. The experimenter looked at the object and said “Modi. A Modi. Modi.” After the naming moment, the objects were then re-presented to the child at the same locations as in Phase 1.

## Supporting Information

S1 Fig The timeline of an individual in experiment 1 (no-switch condition), showing the neural activity in the Vision, Posture, and Word Fields as well as the visual input to iCub at each step. (TIF)

S2 Fig A simplified schematic of the resulting learned network showing the weight values of connections at the point of saying ‘modi’ in step 8 of the network depicted in S1 Fig.
(NOTE only connections with a value greater than 0.05 are shown here.)
(TIF)

S3 Fig Graphs showing the visual activity in step 8 (see S1 Fig.) of four different individual runs in experiment 1 (no-switch condition). **Left: shows a clear success being the same network depicted in S1 and S2 Figs. MidLeft: again shows** a successful object selection despite higher activity in the other object prior to priming. **MidRight:** shows a network which experienced greater interference but in this instance just makes the correct decision. **Right:** a network which selected the other object (this network is also depicted in S4 Fig.).
(TIF)

S4 Fig A simplified schematic of the resulting learned network showing the weight values of connections at the point of saying ‘modi’ in step 8 of the network depicted in S1 Fig.
(NOTE only connections with a value greater than 0.05 are shown here.)
(TIF)

S5 Fig The timeline of an individual in experiment 2 (switch condition), showing the neural activity in the Vision, Posture, and Word Fields as well as the visual input to iCub at each step. (TIF)

S6 Fig Graphs showing the visual activity in step 8 (see S5 Fig.) of four different individual runs in experiment 2 (switch condition). **Left:** shows a clear success being the same network depicted in S5 Fig. **MidLeft:** again shows second individual successful object selection. **MidRight and Right:** show networks that selected the other object.
(TIF)

S7 Fig A simplified schematic of the resulting learned network showing the weight values of connections at the point of saying ‘modi’ in step 8 of the network depicted in S6 Fig. -MidLeft. (NOTE only connections with a value greater than 0.05 are shown here.)
(TIF)

S8 Fig The timeline of an individual in experiment 3 (posture change), showing the neural activity in the Vision, Posture, and Word Fields as well as the visual input to iCub at each step. (TIF)

S9 Fig Graphs showing the visual activity in step 8 (see S8 Fig.) of four different individual runs in experiment 3 (posture change). (TIF)

S10 Fig A simplified schematic of the resulting learned network showing the weight values of connections at the point of saying ‘modi’ in step 8 of the network depicted in S8 Fig.
(NOTE only connections with a value greater than 0.05 are shown here.)
(TIF)

S11 Fig The timeline of an individual in experiment 4 (interference task), showing the neural activity in the Vision, Posture, and Word Fields as well as the visual input to iCub at each step. (TIF)

S12 Fig Graphs showing the visual activity in step 8 (see S11 Fig.) of four different individual runs in experiment 4 (spatial interference task). **Left:** shows a clear success being the same network depicted in S11 Fig. **MidLeft:** again shows second individual successful object selection. **MidRight and Right:** show networks that selected the other object.
(TIF)

S13 Fig A simplified schematic of the resulting learned network showing the weight values of connections at the point of saying ‘modi’ in step 8 of the network depicted in S11 Fig.
(NOTE only connections with a value greater than 0.05 are shown here.)
(TIF)

S14 Fig The timeline of an individual in experiment 5 (interference task with posture change over step 9), showing the neural activity in the Vision, Posture, and Word Fields as well as the visual input to iCub at each step. (TIF)

S15 Fig Graphs showing the visual activity in step 10 (see S14 Fig.) of four different individual runs in experiment 5 (spatial interference task with posture change in step 8). **Left:** shows a clear success being the same network depicted in S14 Fig. **MidLeft:** again shows second individual successful object selection. **MidRight and Right:** show networks that selected the other object.
(TIF)

S16 Fig A simplified schematic of the resulting learned network showing the weight values of connections at the point of saying ‘modi’ in step 8 of the network depicted in S14 Fig. -Left. (NOTE only connections with a value greater than 0.05 are shown here.)
(TIF)

S17 Fig Summary of the robot model results across each experiment, shown for two different learning rates, 0.012 was the best match to child data found, and 0.1. In both cases the same qualitative pattern is seen between each experimental condition demonstrating a qualitative robustness to variations in the learning rate.
(TIF)

S1 File Detailed descriptions and analysis of the robot experiments and results. (DOCX)

S1 Video Video showing the interaction with the robot for experiments 4 (interference task) and 5 (interference with posture change). (MP4)
