# Supplementary figures and images for: Posture Affects How Robots and Infants Map Words to Objects
Source: PLoS One. 2015 Mar 18;10(3):e0116012. doi: 10.1371/journal.pone.0116012 (PMC4364718; doi:10.1371/journal.pone.0116012)

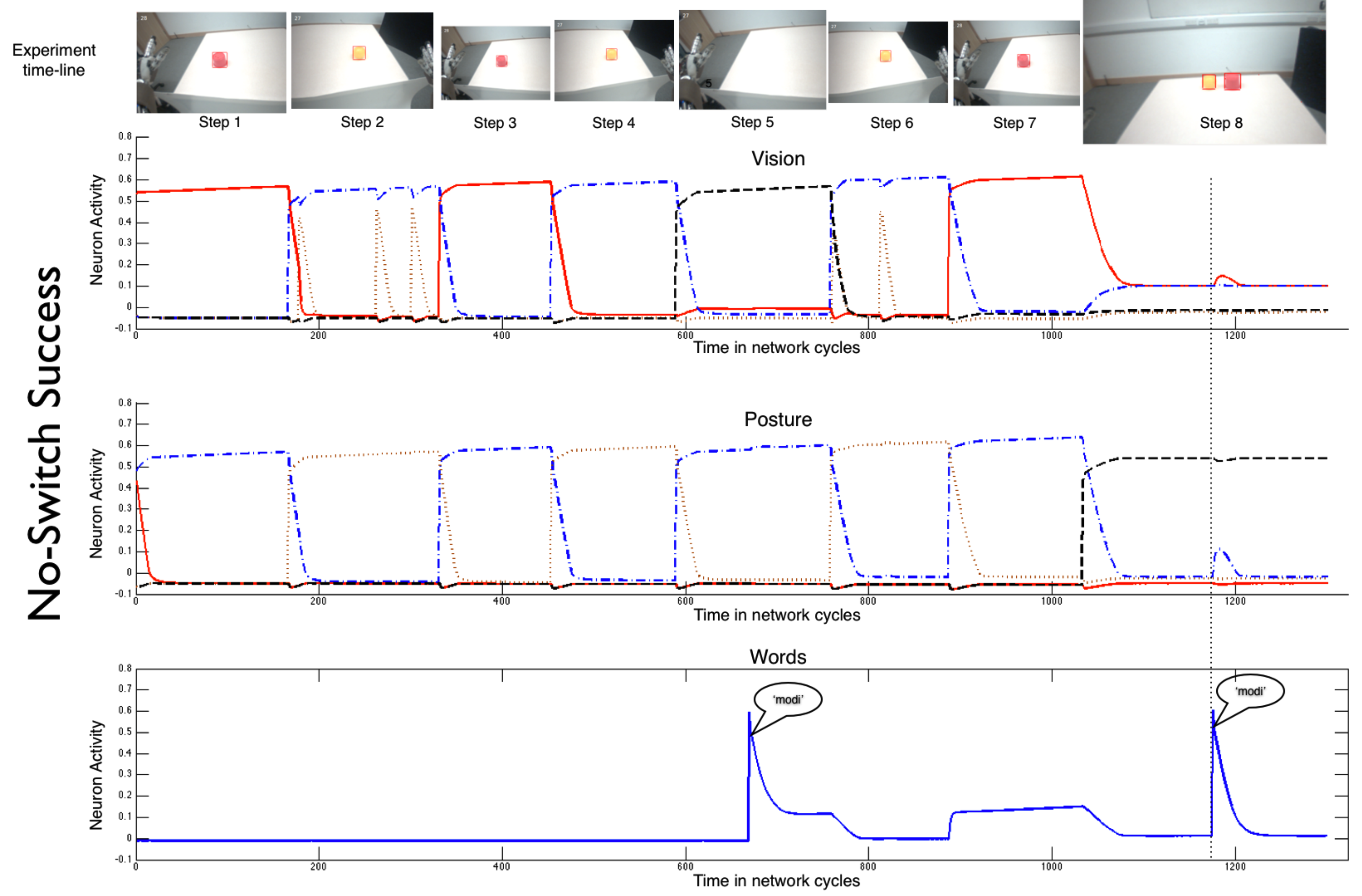

Supplement: S1 Fig — (TIF) [file pone.0116012.s001.tif]

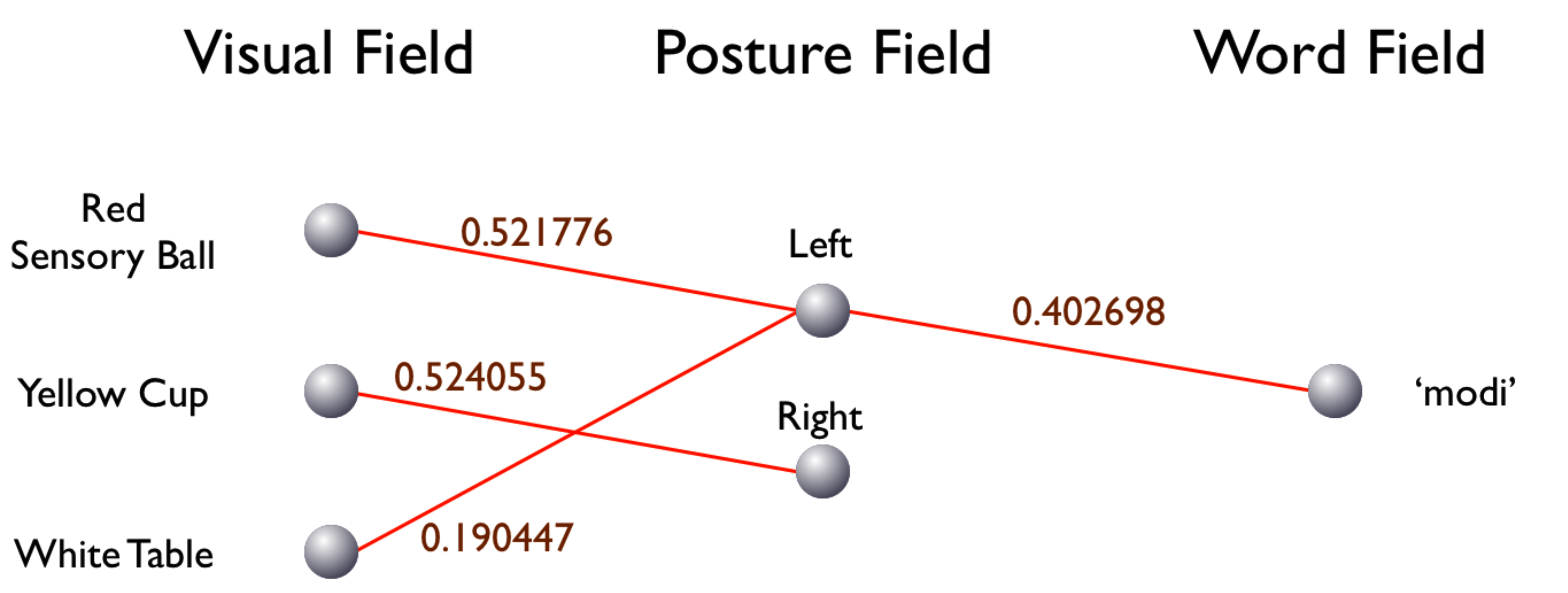

Supplement: S2 Fig — (NOTE only connections with a value greater than 0.05 are shown here.) (TIF) [file pone.0116012.s002.tif]

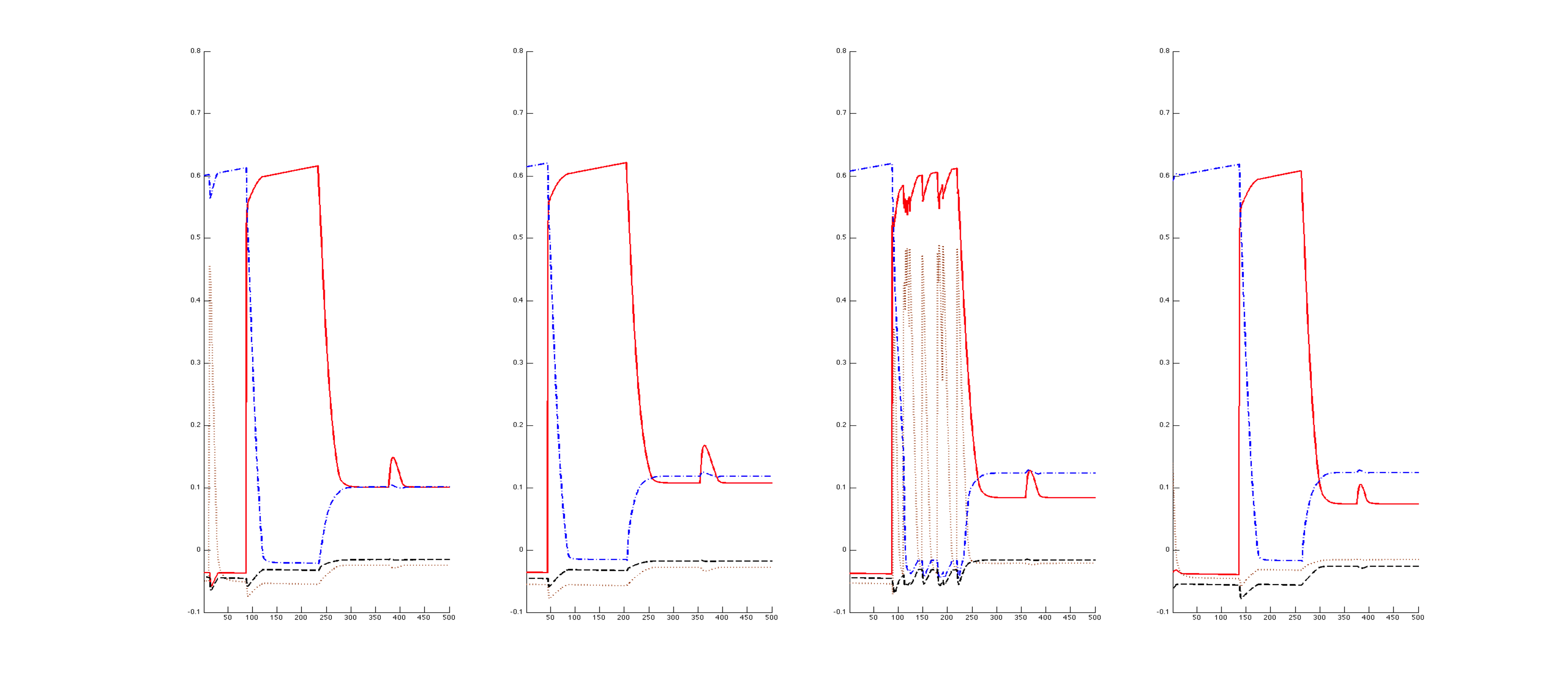

Supplement: S3 Fig — Left: shows a clear success being the same network depicted in S1 and S2 Figs. MidLeft: again shows a successful object selection despite higher activity in the other object prior to priming. MidRight: shows a network which experienced greater interference but in this instance just makes the correct decision. Right: a network which selected the other object (this network is also depicted in S4 Fig.). (TIF) [file pone.0116012.s003.tif]

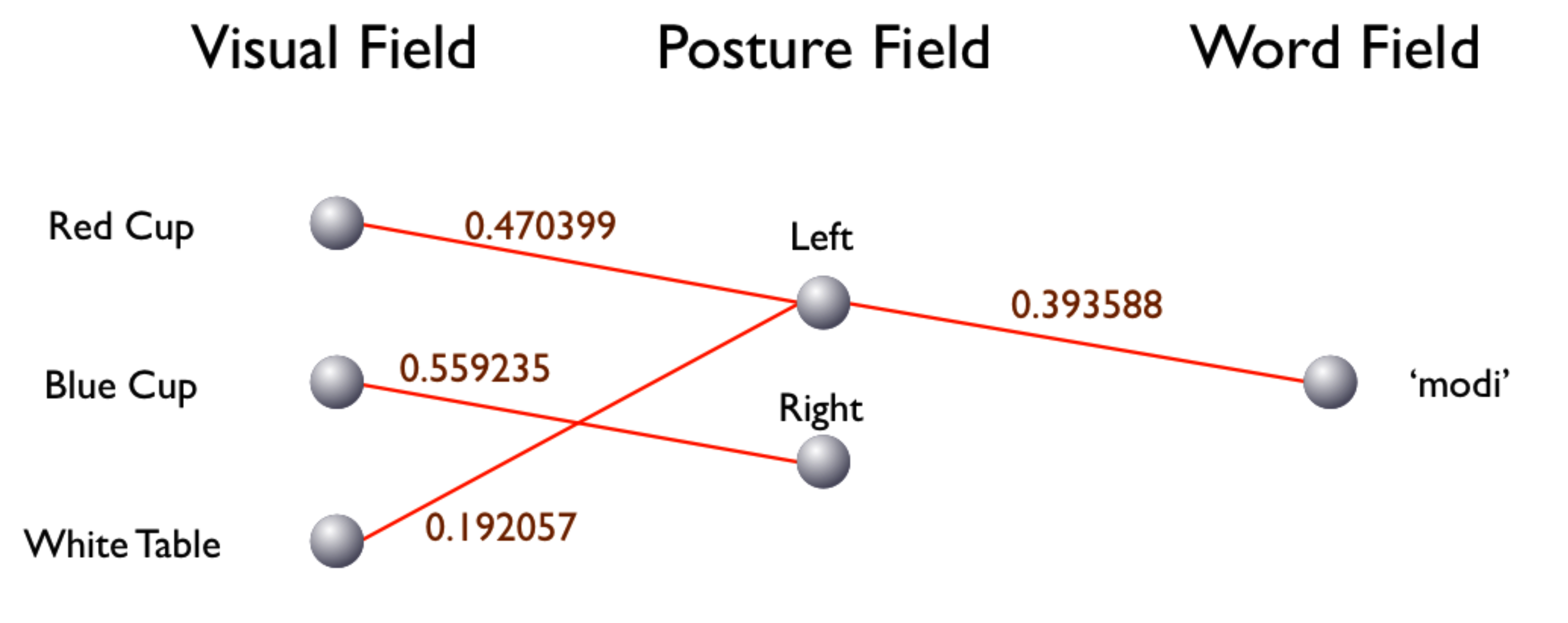

Supplement: S4 Fig — (NOTE only connections with a value greater than 0.05 are shown here.) (TIF) [file pone.0116012.s004.tif]

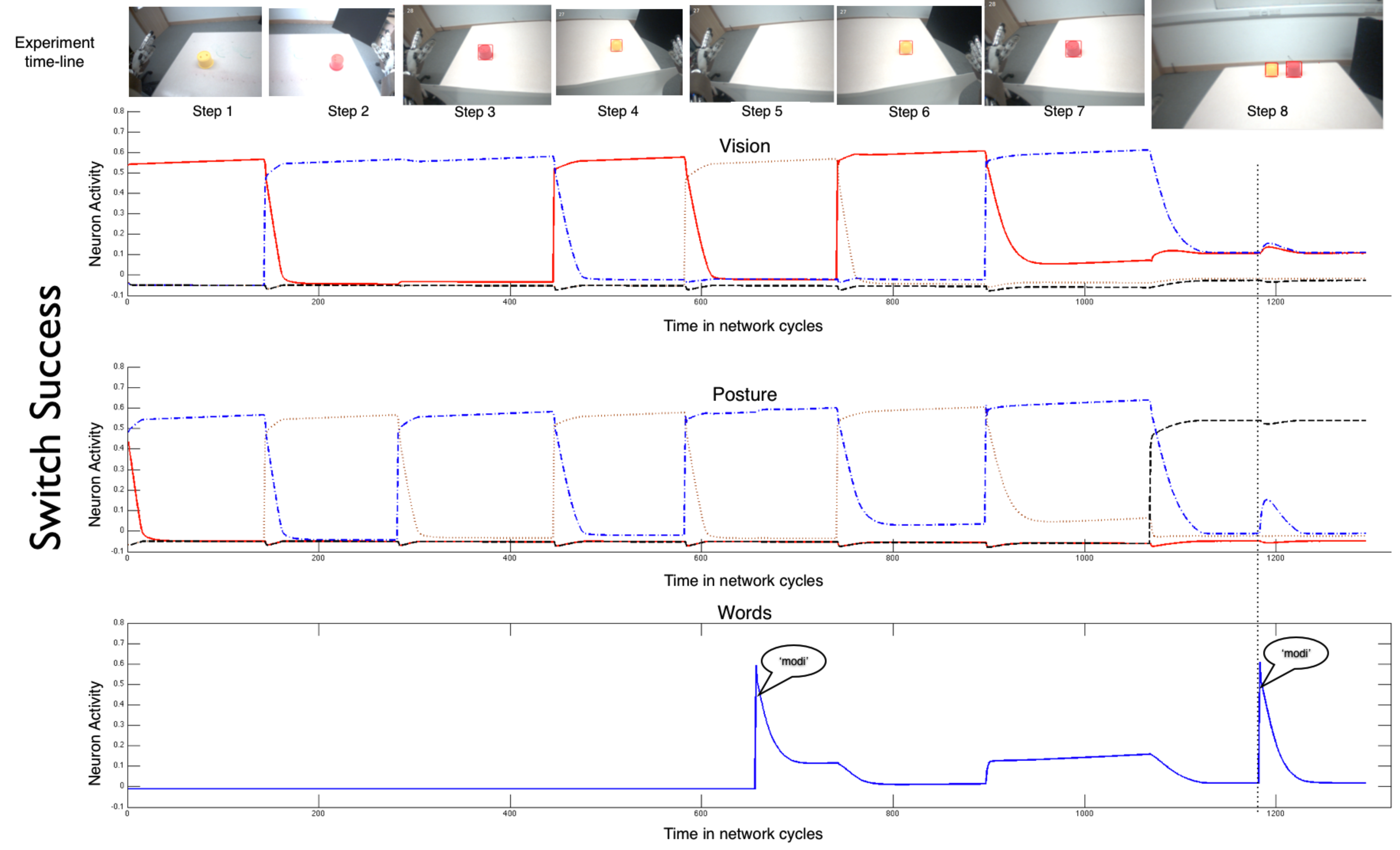

Supplement: S5 Fig — (TIF) [file pone.0116012.s005.tif]

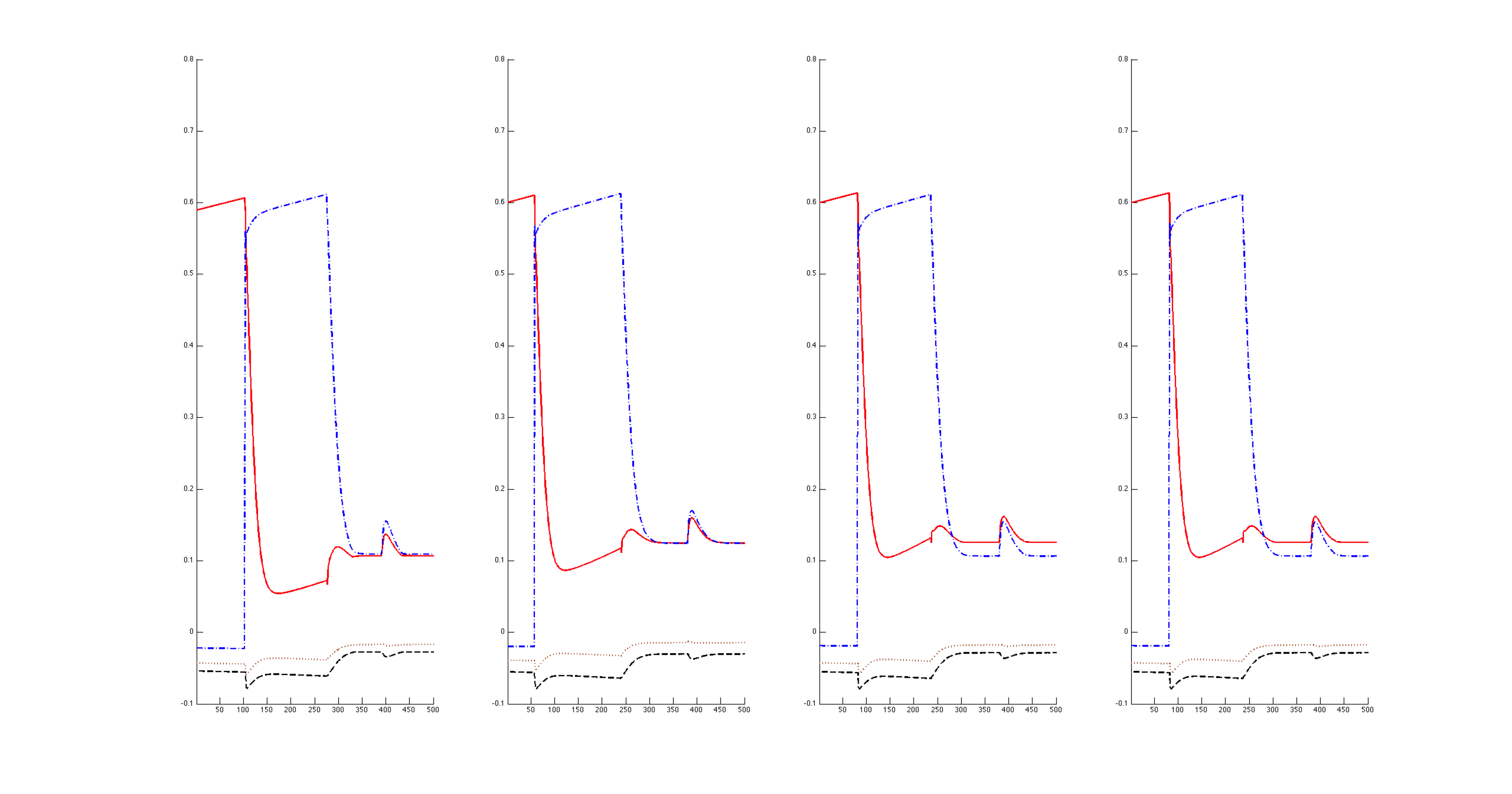

Supplement: S6 Fig — Left: shows a clear success being the same network depicted in S5 Fig. MidLeft: again shows second individual successful object selection. MidRight and Right: show networks that selected the other object. (TIF) [file pone.0116012.s006.tif]

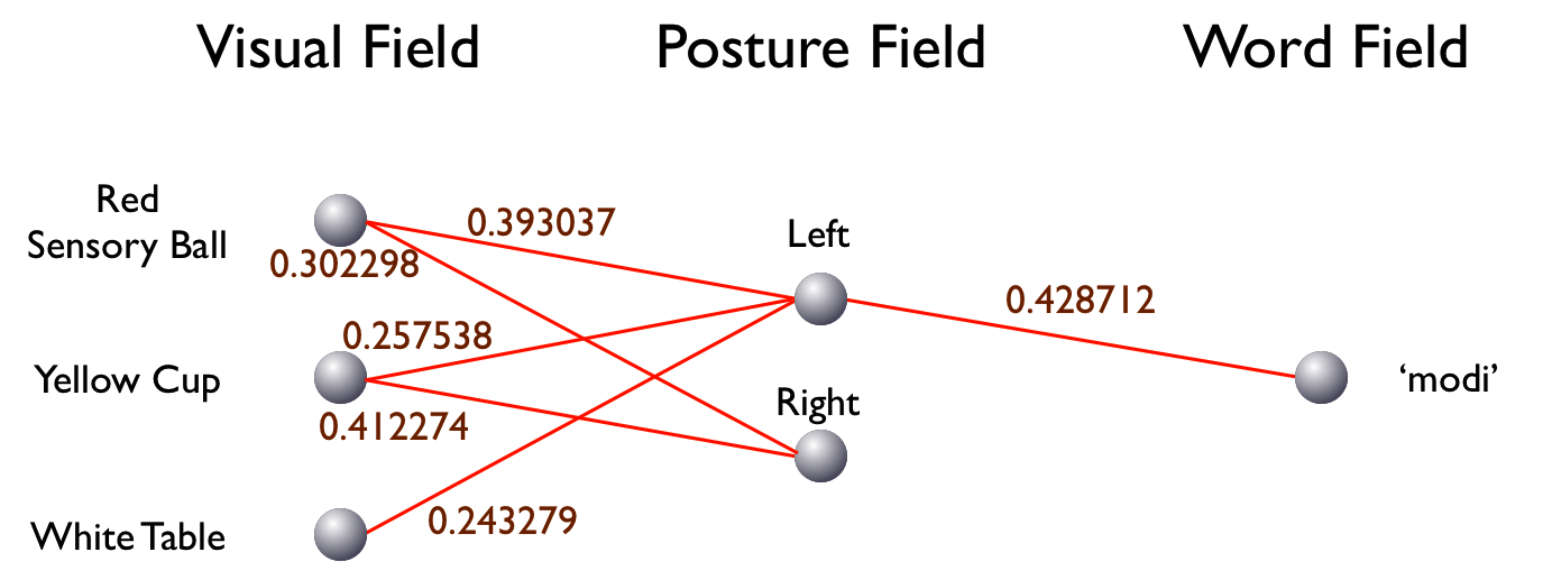

Supplement: S7 Fig — (NOTE only connections with a value greater than 0.05 are shown here.) (TIF) [file pone.0116012.s007.tif]

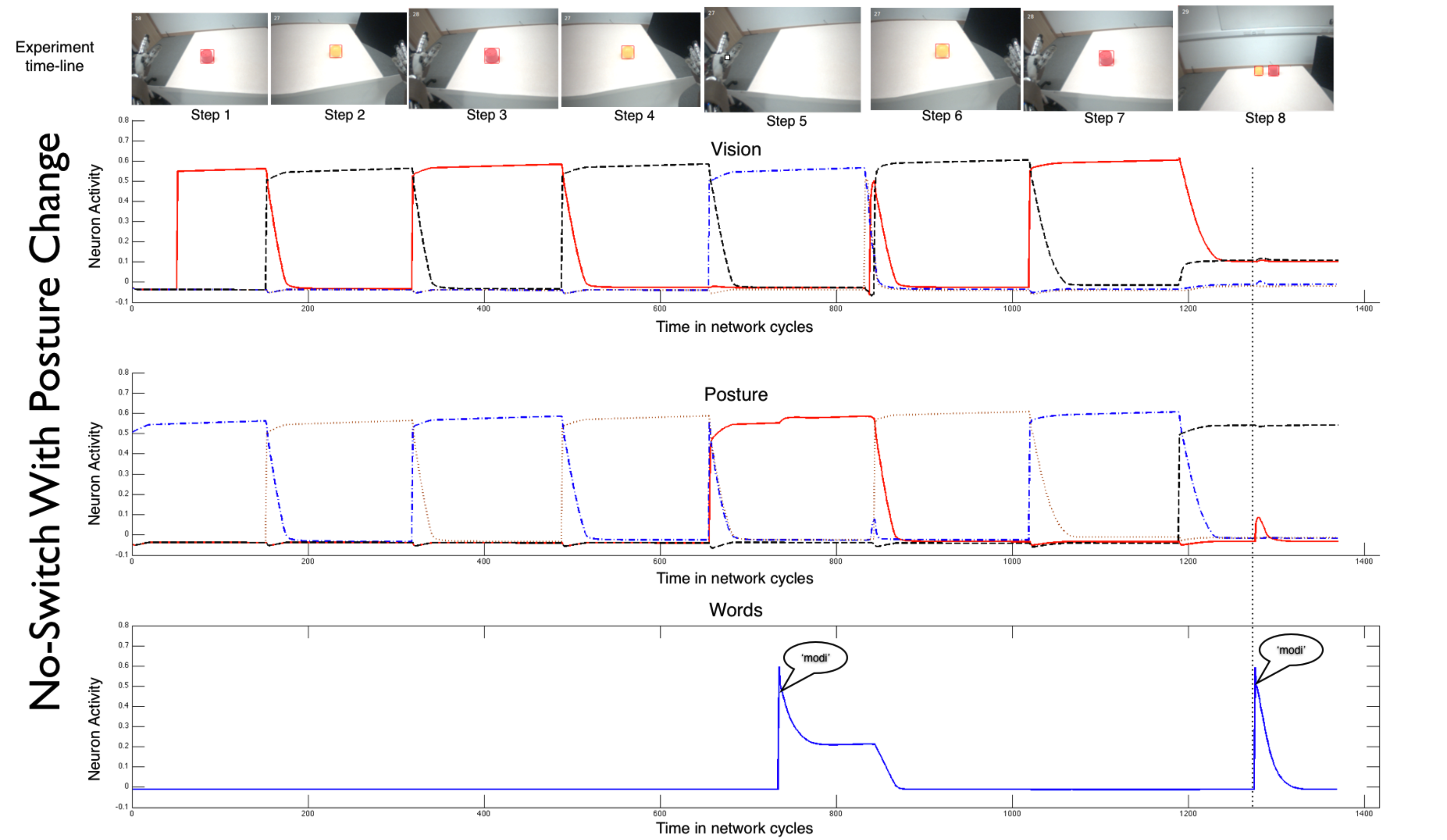

Supplement: S8 Fig — (TIF) [file pone.0116012.s008.tif]

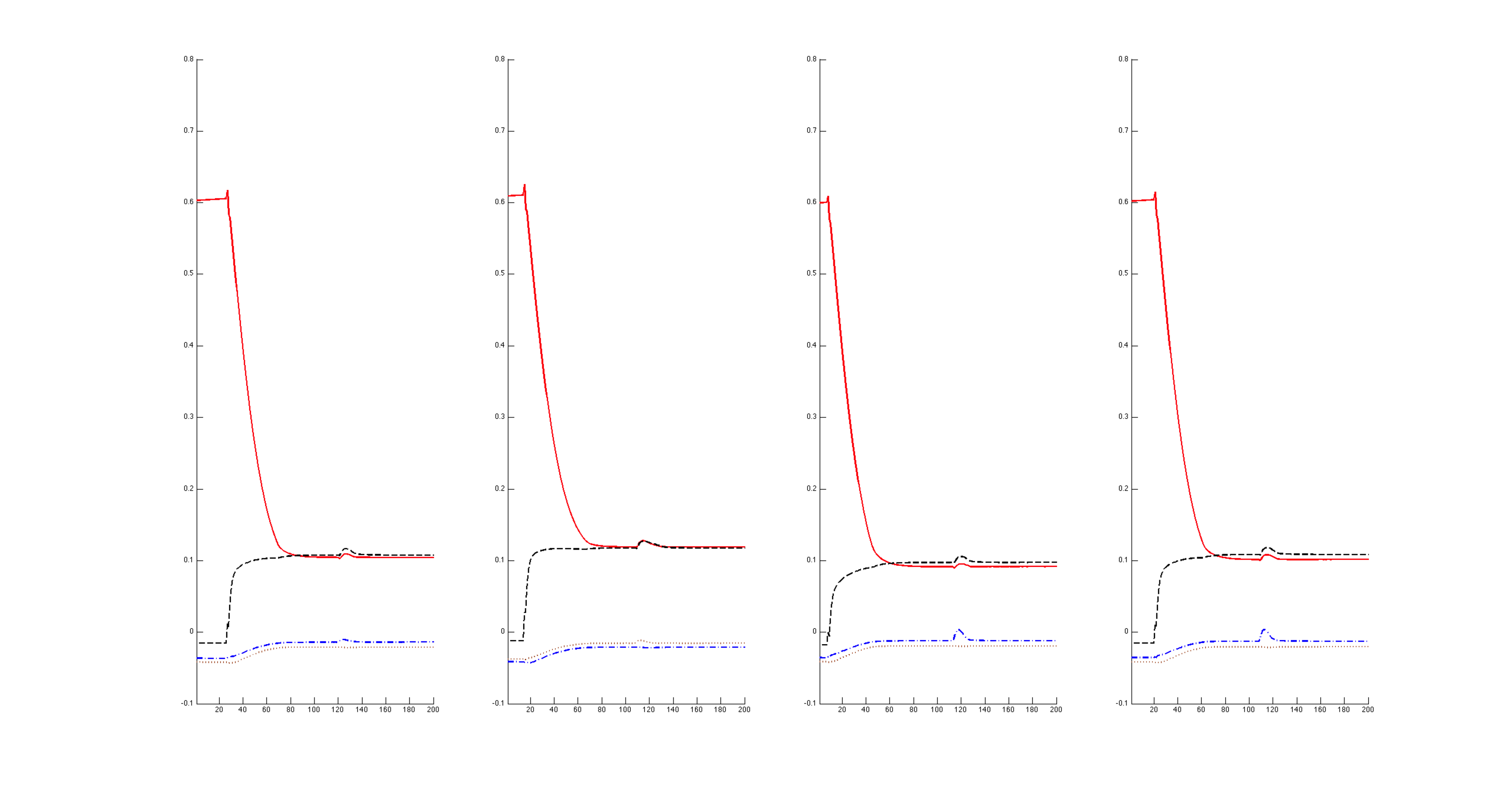

Supplement: S9 Fig — (TIF) [file pone.0116012.s009.tif]

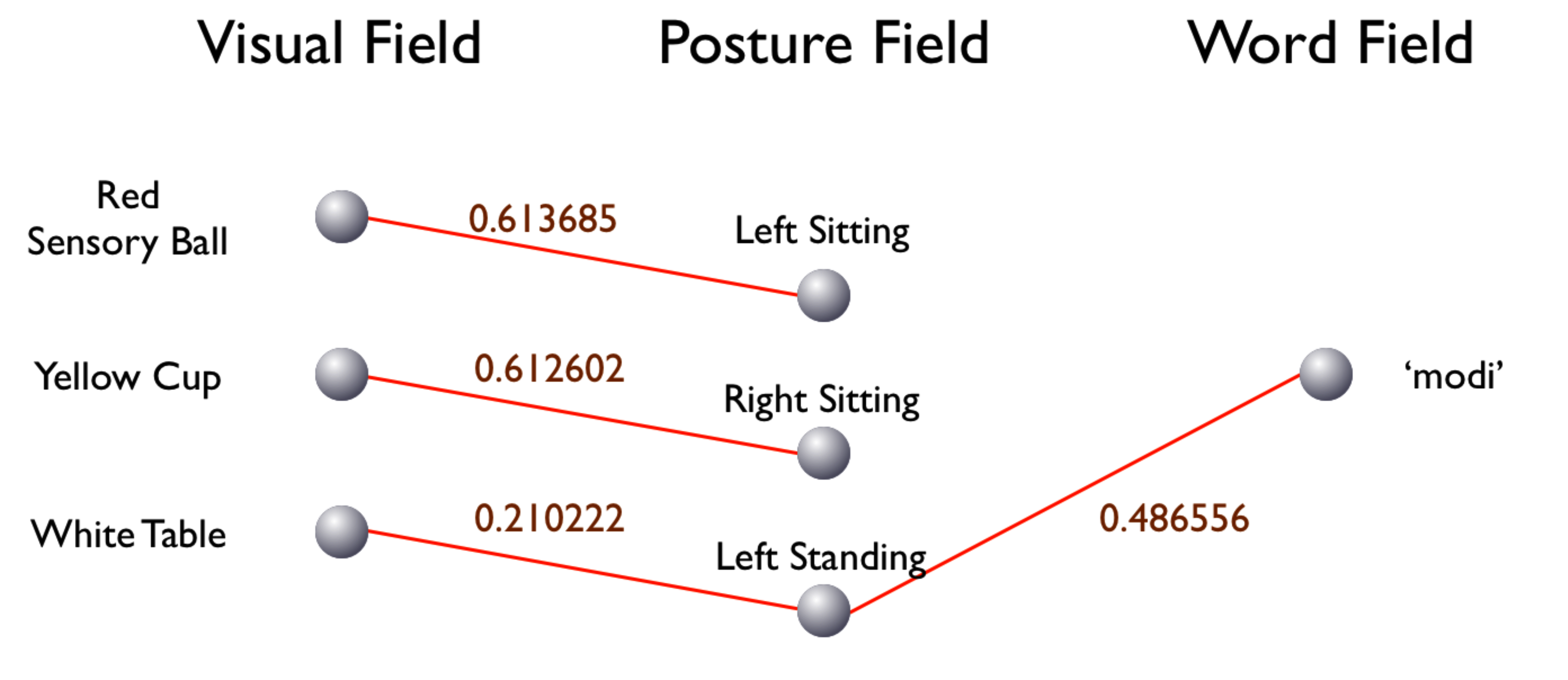

Supplement: S10 Fig — (NOTE only connections with a value greater than 0.05 are shown here.) (TIF) [file pone.0116012.s010.tif]

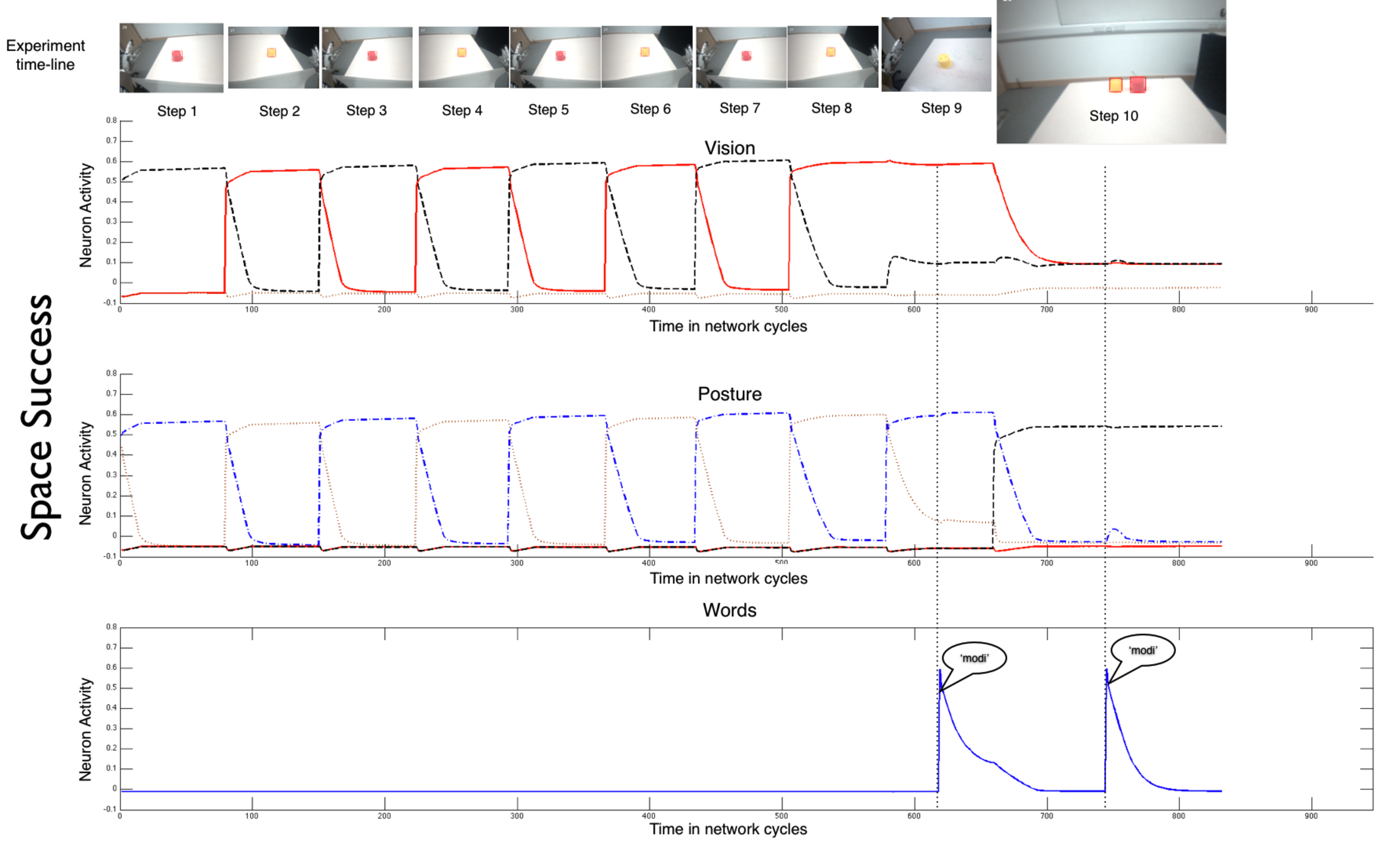

Supplement: S11 Fig — (TIF) [file pone.0116012.s011.tif]

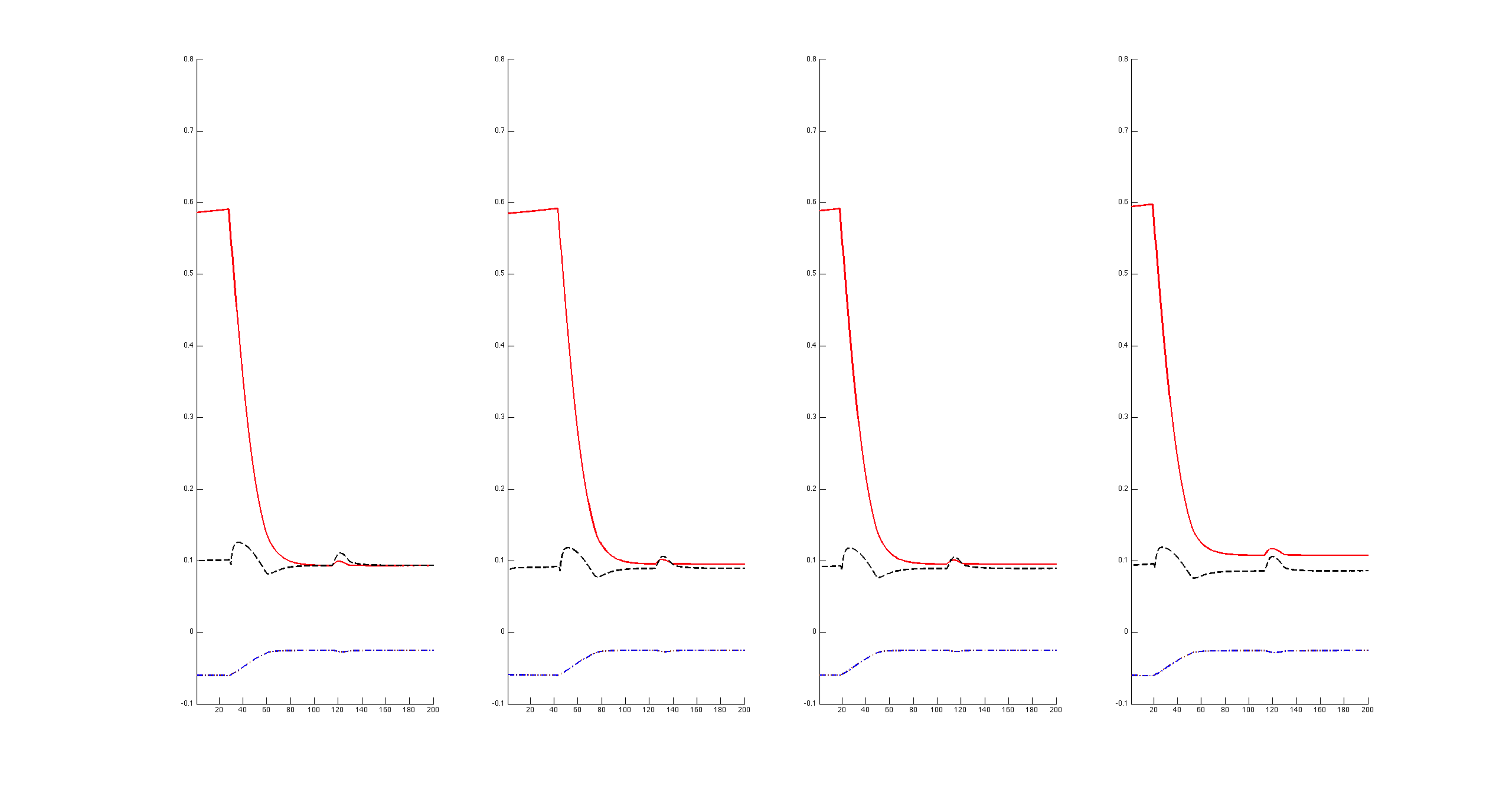

Supplement: S12 Fig — Left: shows a clear success being the same network depicted in S11 Fig. MidLeft: again shows second individual successful object selection. MidRight and Right: show networks that selected the other object. (TIF) [file pone.0116012.s012.tif]

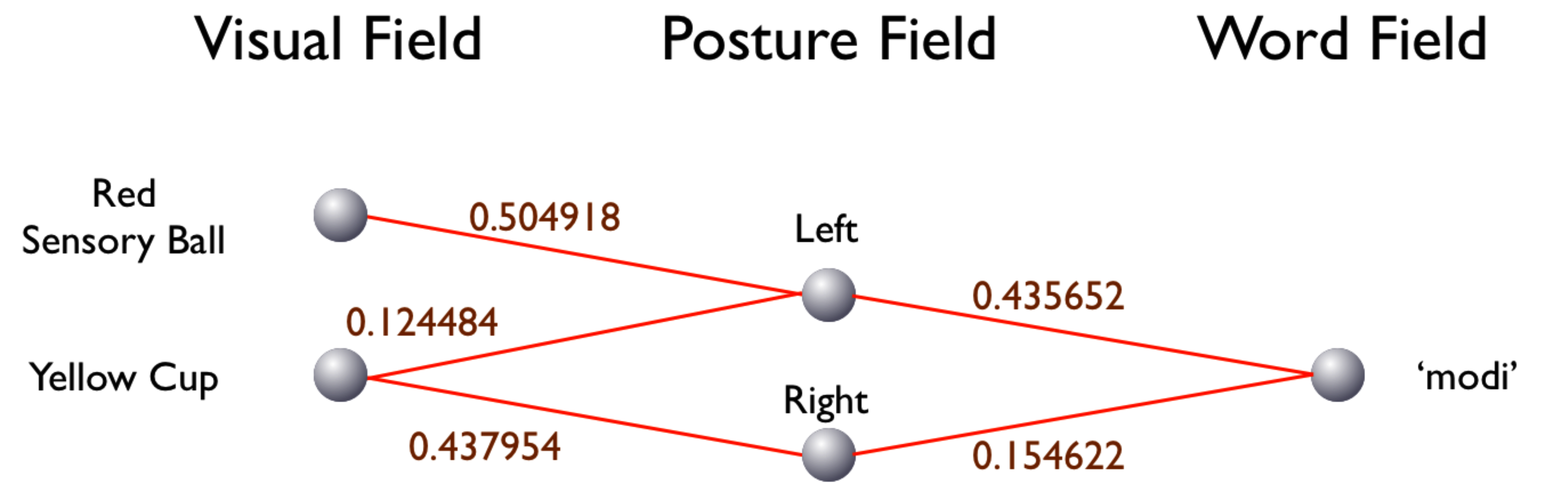

Supplement: S13 Fig — (NOTE only connections with a value greater than 0.05 are shown here.) (TIF) [file pone.0116012.s013.tif]

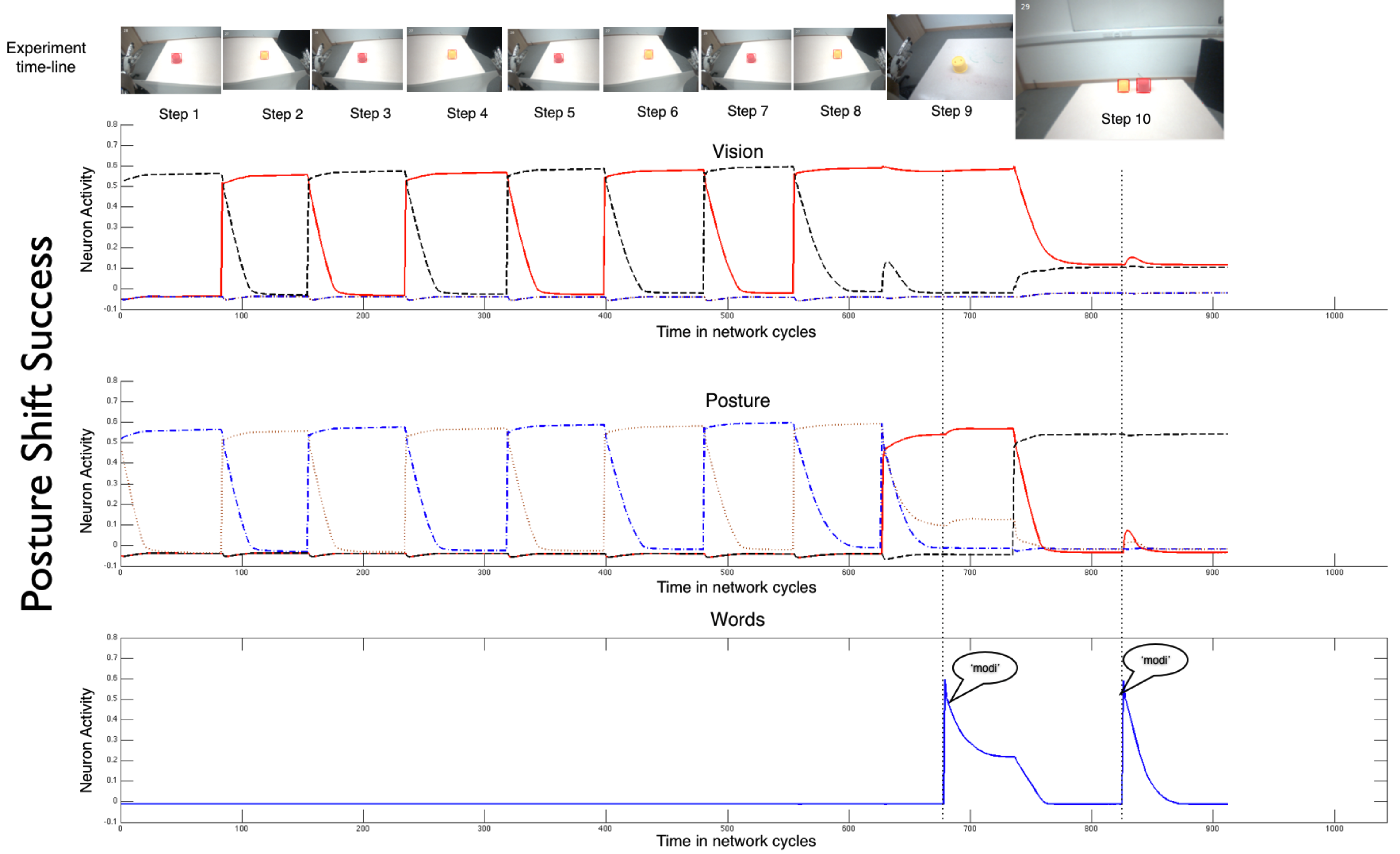

Supplement: S14 Fig — (TIF) [file pone.0116012.s014.tif]

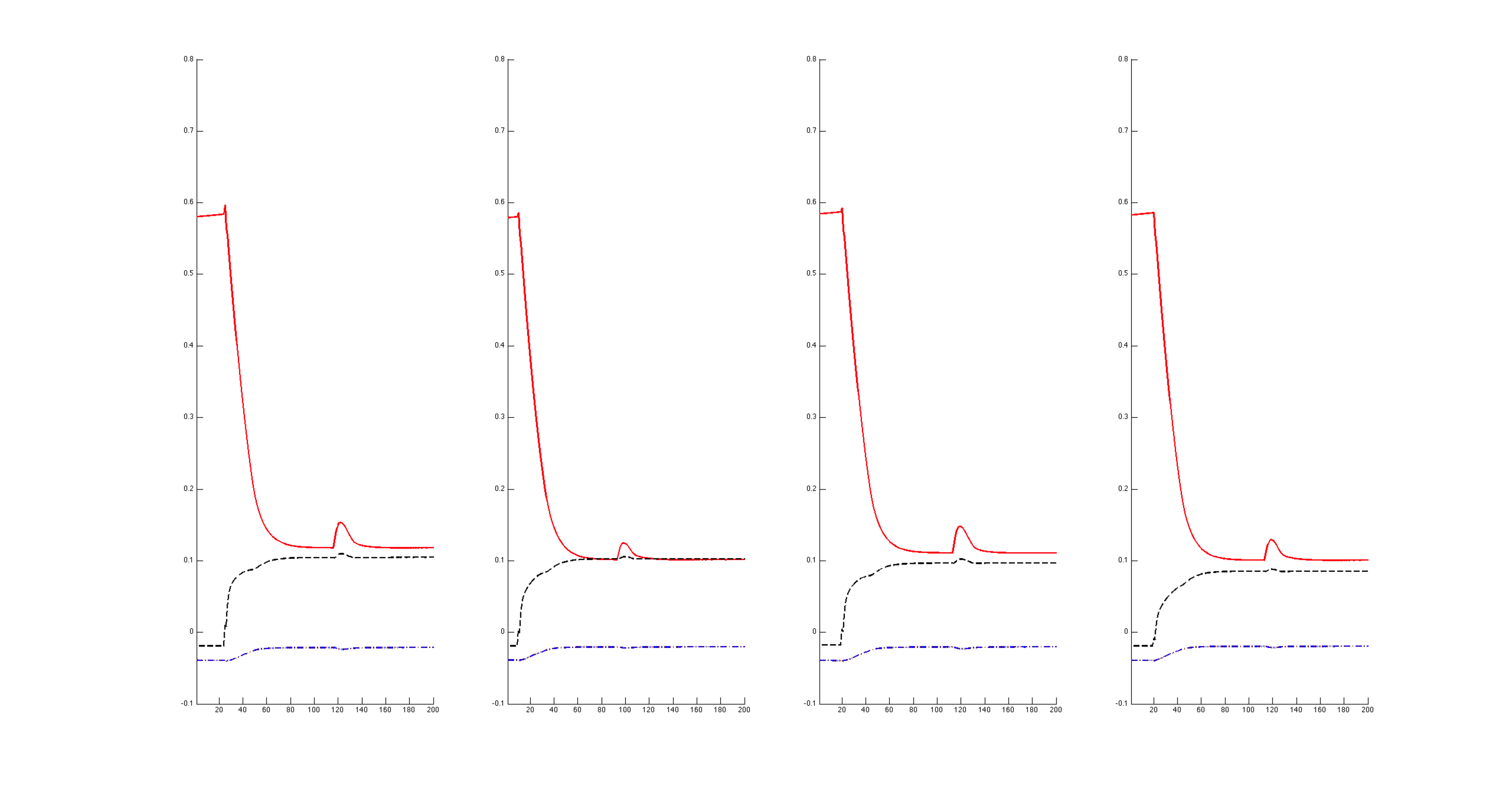

Supplement: S15 Fig — Left: shows a clear success being the same network depicted in S14 Fig. MidLeft: again shows second individual successful object selection. MidRight and Right: show networks that selected the other object. (TIF) [file pone.0116012.s015.tif]

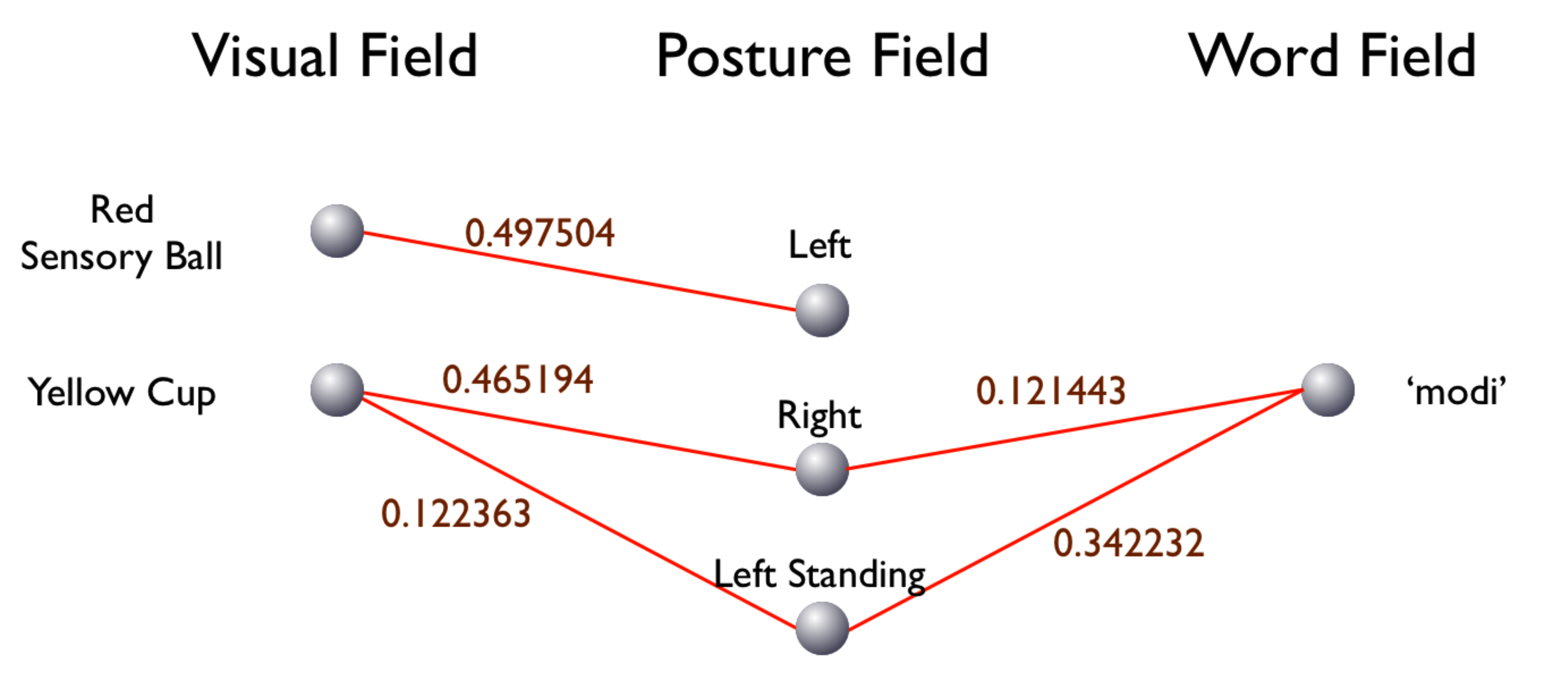

Supplement: S16 Fig — (NOTE only connections with a value greater than 0.05 are shown here.) (TIF) [file pone.0116012.s016.tif]

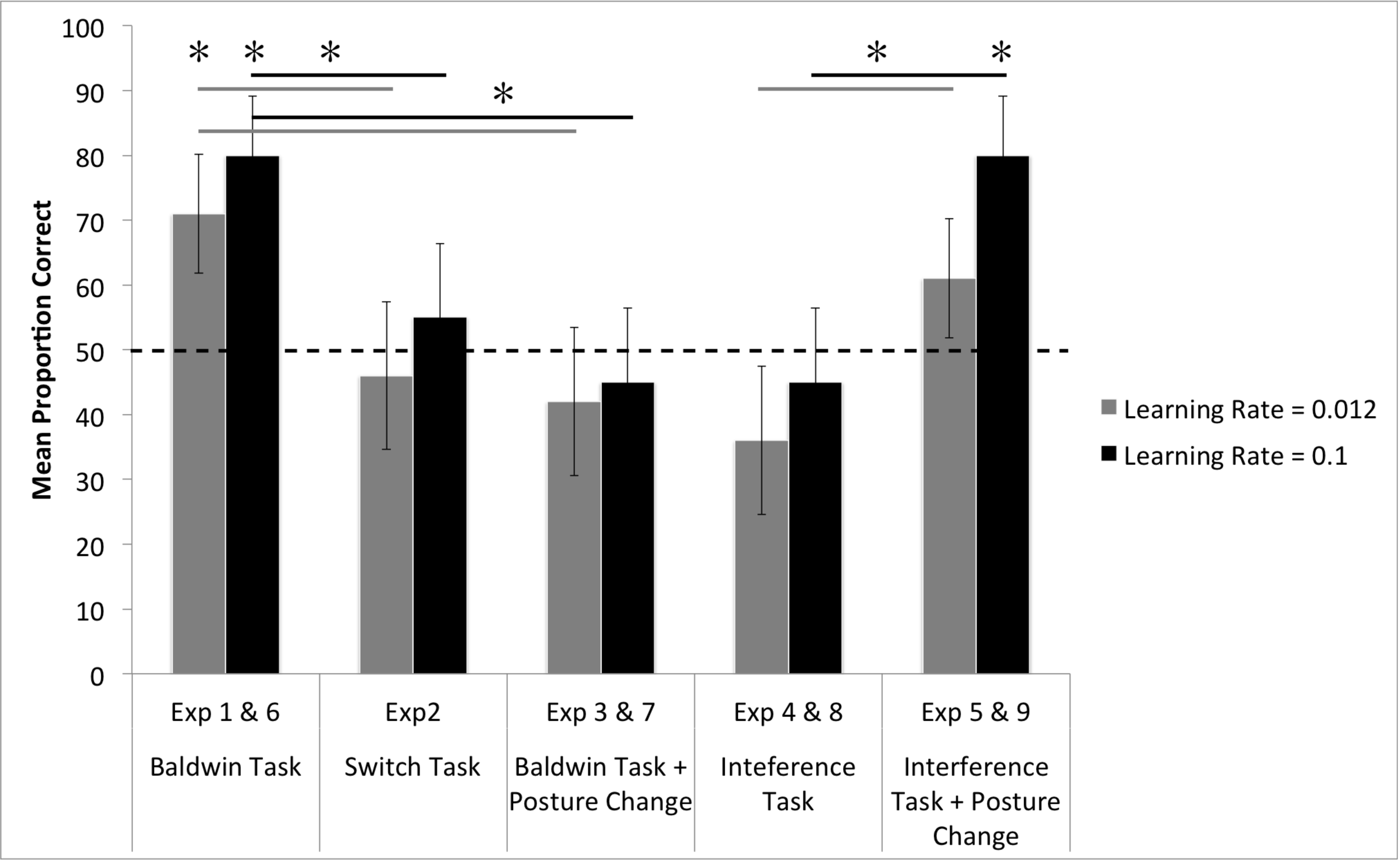

Supplement: S17 Fig — In both cases the same qualitative pattern is seen between each experimental condition demonstrating a qualitative robustness to variations in the learning rate. (TIF) [file pone.0116012.s017.tif]
