# Supplementary material for: Posture Affects How Robots and Infants Map Words to Objects
Source: PLoS One. 2015 Mar 18;10(3):e0116012. doi: 10.1371/journal.pone.0116012 (PMC4364718; doi:10.1371/journal.pone.0116012)
Supplement: S1 File — (DOCX) [file pone.0116012.s018.docx]

Supplementary Information:

1. Details of the computational model:

The computational model builds an associative network between three fields or pools of nodes, two of which are populated by Self-Organizing Maps. The first of these maps receives as input the average RGB color of the fovea region of the left camera of the iCub robot. The fovea is defined as a 10x10 pixel box region in the center of the image. The SOM is a 100 node, 2D map initialized with random values and is partially pre-trained (until the neighborhood size reaches 1) with normalized random RGB values in the range of values discoverable by the camera. Training of the SOM follows the standard equations shown below:

Equation 1: SOM activity

$$BMU=Max_{i}\left(1-\sqrt{\Sigma(a_{j}-w_{ij})}\right)$$

The Best Matching Unit is the unit with weights (*w*) most closely matching the current input vector (*a*),

Equation 2: SOM Learning Rule

$$\Delta w_{ij}=\alpha\exp\left(-\frac{dist^{2}}{2size}\right)(a_{j}w_{ij})$$

The weights of each node within the neighborhood of the BMU are then modified to move closer to the current input vector, scaled according to the distance of this node from the BMU in the space of the SOM (i.e. not in terms of the input space).

The neighborhood size and learning rate both monotonically decrease until the neighborhood size is 1 at which point both the neighborhood size and the learning rate of the SOM is fixed to allow learning to continue at a low rate.

The second SOM receives input from the joint angle encoders of iCub’s eyes, head, and torso (pan and tilt positions for each). As with the color SOM this posture SOM is also partially pre-trained with normalized random input vectors in the range achievable by the various joints.

The third field or pool of nodes is dynamically generated as new words are encountered. Speech input from a microphone is processed by the commercial speech-to-text software ‘Dragon Dictate’. Each word (as text) is then processed and a corresponding node is created in the word pool in an online and ongoing manner such that any previously encountered word will activate the same unit generated when that same word was first encountered.

Following partial pre-training of the two SOMs, the color and word pools are fully connected to the posture pool with weights initially valued at zero. A Hebb-like learning algorithm is then applied to these connections using the following equations:

Equation 3: IAC Spreading Activation

$$net_{i}=\Sigma w_{ij}a_{j}+\beta BMU_{i}$$

If *net_i_ >* 0 $\Delta a_{i}=(\max-a_{i})net_{i}-decay(a_{i}-rest)$

Else $\Delta a_{i}=(a_{i}-\min)net_{i}-decay(a_{i}-rest)$

The net input to each unit in the whole network is the sum of spreading activation plus external activation if this node happens to be the BMU of a SOM or a currently active word. In all experiments the following parameter values were used:

External Input Bias (β in equation 3.1) = 0.5

Max (equation 3.2) = 1

Min (equation 3.3) = -0.2

Decay (equation 3.2 & 3.3) = 0.5

Rest (equation 3.2 & 3.3) = -0.01

Equation 4: Hebb-like Learning Rule

If *a_i_* _OR_ *a_j_* > 0:

if *a_i_ a_j_* > 0 $\Delta w_{ij}=\lambda a_{i}a_{j}(1-w_{ij})$

else $\Delta w_{ij}=\lambda a_{i}a_{j}(1+w_{ij})$

The Hebb-like learning rule increases the strength of a connection if both units connected by this weight are positively active or reduces the strength of the weight if only one is positively active, and scales that change according to the product of their activity and how close the weights are to 1 or -1 respectively for positive and negative weight changes. Finally each field or pool is fully connected by fixed inhibitory connections.

The experiments were repeated twice with 2 different learning rates:

λ = 0.1

λ = 0.012

Comparison of the results achieved with different learning rates is provided in S17 Figure and discussed briefly in the main paper, however as the lower learning rate provided the best match to the infant data the following analysis of network behavior and internal weights is for the lower learning rate only.

This network then forms part of a subsumption architecture connecting to and controlling the iCub robot. Consecutive images from iCub’s left camera are subtracted and threshold to identify areas of change in the image (movement), this change saliency image is then used to drive iCub to orientate its head and eyes so as to center the region of maximum change in its image. This process is stopped while iCub moves. When iCub is asked to ‘find’ something then external input to the color field or pool is stopped temporarily and the primed activity in the color field or pool is used as a filter to find the best match to that color in the current image. This color-filtered image is then thresholded and suppresses the motion saliency image so that iCub now orients its head and eyes to center the primed salient color. If the primed color is not found in the current image then iCub shakes its head (indicating that it cant find the requested object) and returns to using motion saliency.

1. Analysis of the computational model

Experiment 1 – Replication of the original Baldwin experiment (see paper for details)

S1 Figure above shows the timeline of a successful trial in the no-switch condition of the experiment. The three graphs show the activity of units in the Vision field (upper graph), Posture Field (middle graph), and Word Field (lower graph), while the images above the graphs show what iCub can see at each time. During the first presentation of an object we can see a single color unit, and a single posture unit are both strongly active. When we present the second object in a different position (step 2) we can see that these initially active units quickly decay and a different color – posture pair of units become active.

In step 3 we re-present the original object in its original position and we can see that the same posture and color units from step 1 again become active. Similarly in step 4 we re-present the second object in its location and the same units that were active in step 2 become active again. In step 5 we remove the objects but direct iCubs attention to the first location (by waving a hand there). While the same posture node from steps 1 and 3 is now active again, we can see a different color response, this is the response to the color of the table as no object is in view. At this point we say the word ‘modi’ and we can see the activity of a word unit responding to this in the bottom graph. The activity of that word unit quickly decays but because the network is learning and because the same color and posture units are active it doesn’t decay completely. In step 6 we re-present the second object in the second location and we can see the color and posture units change, but we also see the priming of the word unit disappear and the word now decays to a base level. Step 7 re-presents the first object in the first position and now not only do we see the same color and posture response as in steps 1 and 3, but we also see again the word unit being primed as its activity level rises. Finally in Step 8 we draw iCubs attention to a new location and we block visual input because we want to see what color is primed by the word ‘modi’ rather than what color iCub is currently looking at. After a brief pause we ask iCub to find the ‘modi’, we can see the word response is accompanied by an increase in activity of both the primed posture and object, thereby enabling iCub to filter the image for this primed color and select the correct object.

S2 Figure below shows a diagram of the relevant parts of the neural network and the strength of the connecting weights at the point of testing (stage 8), here we should note that the connections between the visual objects and postures have similar values.

S3 Figure shows this final step (step 8) for several different individual runs of the experiment highlighting the extent of individual variation. This variation results from the relative strengths of connections between the visual features and the postures, which vary with both the time spent looking at each object, and the possibility of variation in response due to noise. Even in the highly successful run depicted in S1 Figure, we can see that a small amount of variation in the visual response was present in steps 2 and 6 where a second SOM feature responded causing spikes in that features activity and corresponding dips in the activity of the primarily responding feature. Greater variation in the time spent looking and increased variation in the visual response due to the noise inherent in the camera images resulted in individual variation corresponding to the overall success rate of infants in the same experiment.

S4 Figure below shows a diagram of the relevant parts of the neural network and the strength of the connecting weights at the point of testing (stage 8) for the network which selected the wrong object (see S3 Figure Right), here we should note that the connections between the visual objects and postures have dissimilar values, the connection between the ‘blue cup’ and the right posture being stronger than the connection between the ‘red cup’ and the left posture.

Experiment 2 – Baldwin Switch condition

S5 Figure below shows an individual run in experiment 2 (switch condition), the explanation is the same as for S1 Figure however here we can see that the position of the objects was inverted in steps 1 and 2. Because each object has been encountered in both locations (left and right), the word ‘modi’, whilst priming the correct location, indirectly primes both colors. Despite this the correct object is still more strongly primed in step 8 and so the spatially related object is selected.

S6 Figure shows that while both colors are now primed in this condition the model is more susceptible to making an incorrect decision due to variations in the time spent looking and variations in the effect of camera noise. S7 Figure shows the network connectivity learned during the switch condition of the MidLeft model depicted in S6 Figure.

Experiment 3 – Baldwin experiment with posture change

Experiment 3 repeats the Baldwin study replicated in experiment 1 but now with a posture change from sitting to standing or vice versa in the labeling step (step 5). As we can see in S8 Figure both the posture response and the visual response in step 5 are different to any of the previous steps. As a result in the final testing (step 8) the word ‘modi’ primes the novel posture introduced in step 5 but fails to significantly prime either of the objects and so selection is at chance levels.

S9 Figure shows four individual runs of experiment 3 and by comparison to S3 Figure from experiment 1 we can see that levels of priming are greatly reduced by the addition of a posture change in step 5.

In S10 Figure we can see that the resulting network has no direct or indirect connection between the word and either of the objects.

Experiment 4: Spatial Interference

In Experiment 4 both the target and foil objects are presented in consistent locations four times (steps 1-8) before the target object is placed and labeled in the position associated with the foil object (step 9). In step 9 we can see that not only is the object priming the previously associated posture, but the current posture is also priming the foil object.

At step 10, when visual input is removed and a new posture assumed, the request to find the ‘modi’ primes both objects, though typically the target object is more strongly primed, as can be seen from S12 Figure below. As the difference between primed activities is far less than in experiments 1 and 2, this condition is much more susceptible to interference due to time spent looking and camera noise, resulting in individual differences which again closely resemble that of infants in this experiment condition.

S13 Figure shows that the yellow cup has been associated with both the left and right postures, though more strongly with the right, as this is the posture in which the object was most frequently observed. While the word ‘modi’ was only encountered in the right posture, this was while looking at the yellow cup, which also primed the left posture, and so an association has formed between both left and right postures and the word ‘modi’. Subsequently the word modi primes both the left and right posture (though the left posture more strongly), and so both objects are primed.

Experiment 5: Posture Shift

Experiment 5 replicates experiment 4 but now adds a new posture in step 9 where labeling occurs; here the iCub robot is instructed to stand-up or sit-down. As a result, in step 9, while the target object primes the previously associated posture, the current posture does not prime the foil object (compare the upper graph in step 9 with that shown in S11 Figure).

Due to this difference in Step 9, when iCub is asked to find the ‘modi’ in step 10 there is a much more striking difference between the priming levels of the target and foil object (see S15 Figure), one that is much more robust to timing and noise effects and so a more consistent response is observed between individuals than in experiment 4 where no posture change occurs in step 9.

S16 Figure show the resulting network connectivity of the individual run depicted in S14 Figure. Here we can see that the yellow cup has been associated with both the sitting-right posture and the standing left posture, though more strongly with the sitting right as this posture-object combination was encountered more frequently. The red sensory ball however has only been encountered in the sitting-left posture and so is only associated with that posture. The word ‘modi’, though only encountered in the left-standing posture was simultaneously present with the yellow cup, which primed the sitting right posture, and so associations have been formed between these two postures. The word ‘modi’ therefore primes both sitting right and standing left postures but as these both only prime the yellow cup then there is a clear winner in priming.

Analysis of Robot Experiment Results

Each experimental condition was run 20 times, counterbalancing the position of the target / foil in the left / right postures, and for the posture change experiments counterbalancing the sit then stand Vs stand then sit posture changes. At the end of each experiment the Robot iCub was asked to find the ‘modi’ 4 times and we recorded which object each individual run selected (Target or Foil).

A comparison of the different learning rates is shown below in S17 Figure showing that the higher learning rate consistently produced a higher rate of selection of the target in every experimental condition but retained the qualities differences between conditions.
